# Effects of photobiomodulation combined with rehabilitation exercise on pain, physical function, and radiographic changes in mild to moderate knee osteoarthritis: A randomized controlled trial protocol

**DOI:** 10.1371/journal.pone.0314869

**Published:** 2025-01-21

**Authors:** Yan Ling Tay, Mohd Azzuan Ahmad, Nor Hamdan Mohamad Yahaya, Devinder Kaur Ajit Singh

**Affiliations:** 1 Physiotherapy Programme & Centre for Rehabilitation and Special Needs Studies (iCaRehab), Faculty of Health Sciences, Universiti Kebangsaan Malaysia, Kuala Lumpur, Malaysia; 2 Department of Orthopaedic and Traumatology at Faculty of Medicine, Universiti Kebangsaan Malaysia Medical Centre, Kuala Lumpur, Malaysia; 3 Physiotherapy Programme & Center for Healthy Aging and Wellness (H-CARE), Faculty of Health Sciences, Universiti Kebangsaan Malaysia, Kuala Lumpur, Malaysia; Monash University, AUSTRALIA

## Abstract

**Background:**

Photobiomodulation, specifically high-energy photobiomodulation therapy (H-PBMT), is gaining recognition as a promising non-invasive intervention for managing knee osteoarthritis (KOA). While H-PBMT has demonstrated effectiveness in reducing pain and improving physical function, most evidence to date focuses on short-term symptomatic relief. The potential for H-PBMT to offer sustained benefits and modify the underlying progression of KOA remains insufficiently explored, warranting further investigation.

**Objective:**

This study aims to assess the short-term and sustained effects of H-PBMT combined with rehabilitation exercises in patients with mild to moderate KOA, focusing on knee radiographic morphological changes over a 3-month follow-up period.

**Methods:**

This protocol outlines a parallel-group, randomized, double-blind, placebo-controlled trial. Fifty participants with mild to moderate KOA (based on the Kellgren-Lawrence classification) will be randomly assigned to either the active H-PBMT plus exercise group (H-PBMT+E, n = 25) or the placebo photobiomodulation plus exercise group (PL+E, n = 25). Both groups will undergo an 8-week intervention, consisting of conventional rehabilitation exercises paired with either active or placebo photobiomodulation. H-PBMT will be delivered using the BTL-6000 HIL device with a 1064 nm wavelength, providing a total energy dose of 3190 J per 15-minute session. The treatment protocol includes both pulse mode (25 Hz, 5 W, 190 J) for analgesia and continuous mode (5 W, 3000 J) for biostimulation. Participants will be blinded to their group allocation through the use of a placebo device that mimics the active treatment without emitting therapeutic energy. Additionally, the outcome assessors will be blinded to the group allocations to ensure unbiased evaluation of the trial outcomes. The primary outcome is the Knee Injury and Osteoarthritis Outcome Score. Secondary outcomes include the Timed Up-and-Go test, Numerical Pain Rating Scale, and knee X-rays. Outcomes will be evaluated at baseline, immediately post-intervention (week 8), and at 3-month follow-up (week 20). Data will be analyzed according to the intention-to-treat principle, with a two-way repeated measures ANOVA used to assess time, group, and interaction effects.

**Conclusion:**

This study is expected to provide valuable insights into the sustained effects and potential disease-modifying properties of combining H-PBMT with rehabilitation exercises in managing KOA. The findings could inform more effective treatment protocols, improving rehabilitation outcomes and patient quality of life.

**Trial registration:**

Australian New Zealand Clinical Trials Registry (ACTRN12624000699561p).

## Introduction

Knee osteoarthritis (KOA), commonly referred to as degenerative joint disease, is a progressive condition characterized by the gradual degradation and loss of articular cartilage, primarily due to mechanical wear and tear [1]. This degeneration not only compromises the joint’s structural integrity but also leads to substantial functional impairments, manifesting as chronic knee pain, restricted joint range of motion, muscle weakness, gait abnormalities, and impaired balance [1, 2]. These symptoms collectively contribute to reduced mobility and overall disability in affected individuals [1, 2]. Radiographic imaging, particularly X-rays, remains the primary diagnostic tool for KOA, providing critical insights into the severity of the disease through detailed visualization of structural changes, such as cartilage loss, subchondral bone sclerosis, joint space narrowing, and osteophyte formation [3, 4]. These radiographic features are commonly assessed using the Kellgren-Lawrence grading scale, a standardized system for evaluating KOA severity based on distinct radiographic markers [3]. The integration of radiographic findings with clinical assessments is essential for a comprehensive understanding of disease progression and for tailoring effective management strategies.

Recent epidemiological data highlights the global burden of KOA as a significant public health concern [5, 6]. A systematic analysis by Cui et al. (2020), examining 56 studies on symptomatic KOA, compared to 19 on radiographic KOA and 3 on self-reported KOA, estimated that symptomatic KOA affects approximately 16% of the global population, with an incidence rate of 203 cases per 10,000 person years [5]. This prevalence underscores KOA’s substantial impact on individual well-being and healthcare systems worldwide, emphasizing the urgent need for effective management strategies [6]. Current KOA management follows a multifaceted approach aimed at alleviating symptoms, enhancing physical function, and improving quality of life [6, 7]. Rehabilitation exercises, particularly aerobic and muscle strengthening programs tailored to patient needs, consistently demonstrate efficacy in reducing pain and enhancing physical function in KOA patients [8, 9]. Additionally, complementary interventions such as electrophysical agents including thermal modalities [10], therapeutic ultrasound [11], and photobiomodulation therapy (PBMT) [12, 13] are frequently incorporated into treatment plans to further enhance outcomes.

Among complementary therapies for KOA, photobiomodulation, specifically high-energy photobiomodulation therapy (H-PBMT), has gained attention as a promising non-invasive intervention with multifaceted therapeutic effects [12, 14]. H-PBMT, also termed high-intensity laser therapy, is noted for its potential to accelerate healing, stimulate tissue regeneration, reduce inflammation, and alleviate pain through its interaction with cellular and tissue components, which triggers photobiomodulatory effects [15]. The rationale for employing H-PBMT in KOA management is reinforced by a growing body of clinical evidence, including systematic reviews and meta-analyses that emphasize its efficacy in pain relief, functional enhancement, and disability reduction, especially in the short term [13, 16, 17]. For example, a study by Song et al. (2020) which analysed six randomized controlled trials reported significant improvements in pain and physical function among KOA patients treated with H-PBMT [17]; however, heterogeneity in study methodologies and sample sizes suggests the need for cautious interpretation of these findings [17]. A recent meta-analysis by Fernandez et al. (2023) affirms H-PBMT as a viable intervention for reducing pain and improving function in musculoskeletal disorders, including KOA, supporting its broader application in clinical practice [14]. Additionally, Ahmad et al. (2022) highlighted that H-PBMT, particularly when combined with exercise therapy and administered at energy densities ranging from 15–810 J/cm^2^ with total doses of 1250–3000 J per session, effectively reduces pain and stiffness in KOA patients [16]. This growing evidence base underscores the relevance of H-PBMT as an adjunctive therapy in managing KOA symptoms, presenting an opportunity for its integration into standardized care protocols.

**Fig 1:**
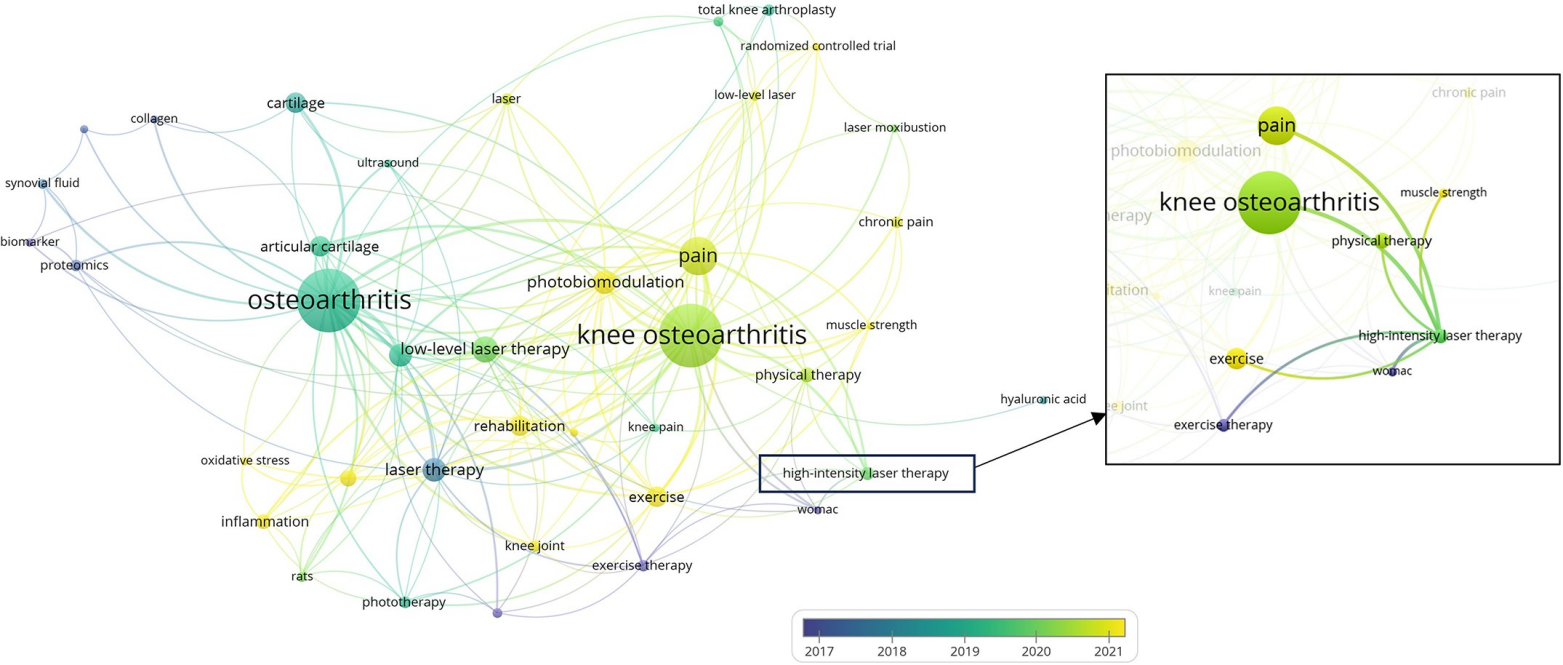
Overview of the research landscape on photobiomodulation therapy for knee osteoarthritis.

Furthermore, emerging evidence indicates that H-PBMT may offer benefits beyond symptomatic relief by supporting radiographic and structural improvements in KOA [13, 18]. Primorac et al. (2023) demonstrated that H-PBMT enhances cartilage metabolism, stimulates collagen production, and reduces synovial inflammation [18]. These effects collectively contribute to slowing cartilage degradation and preserving joint structure [13, 18]. Additionally, animal research using rat models highlights the potential of PBMT in promoting cartilage recovery and controlling central sensitization associated with chronic KOA, which is critical for long-term pain management [19]. Despite these promising findings, the clinical evidence specifically supporting structural benefits of H-PBMT in humans remains limited [18], underscoring the need for further investigation. While considerable research has focused on symptom management, primarily pain reduction and functional improvement through interventions like low-energy PBMT [14, 16], there is a noticeable gap in understanding H-PBMT’s impact on radiographic outcomes and structural modifications within the knee joint (Fig 1).

The scarcity of high-quality evidence supporting the long-term efficacy of H-PBMT, as noted in current treatment guidelines [8, 20, 21], limits its integration into mainstream KOA management. This gap highlights the need for rigorous trials on the sustained effects of H-PBMT, not only on symptomatic relief but also on potential structural benefits in KOA. This study aims to address this need by evaluating the sustained effects of H-PBMT combined with rehabilitation exercises on pain, physical function, and knee joint structure in patients with mild to moderate KOA. The findings may support H-PBMT as a novel, non-invasive treatment that offers both symptomatic relief and structural benefits, contributing to a more comprehensive approach to KOA management.

## Materials and methods

### Study design

**Fig 2:**
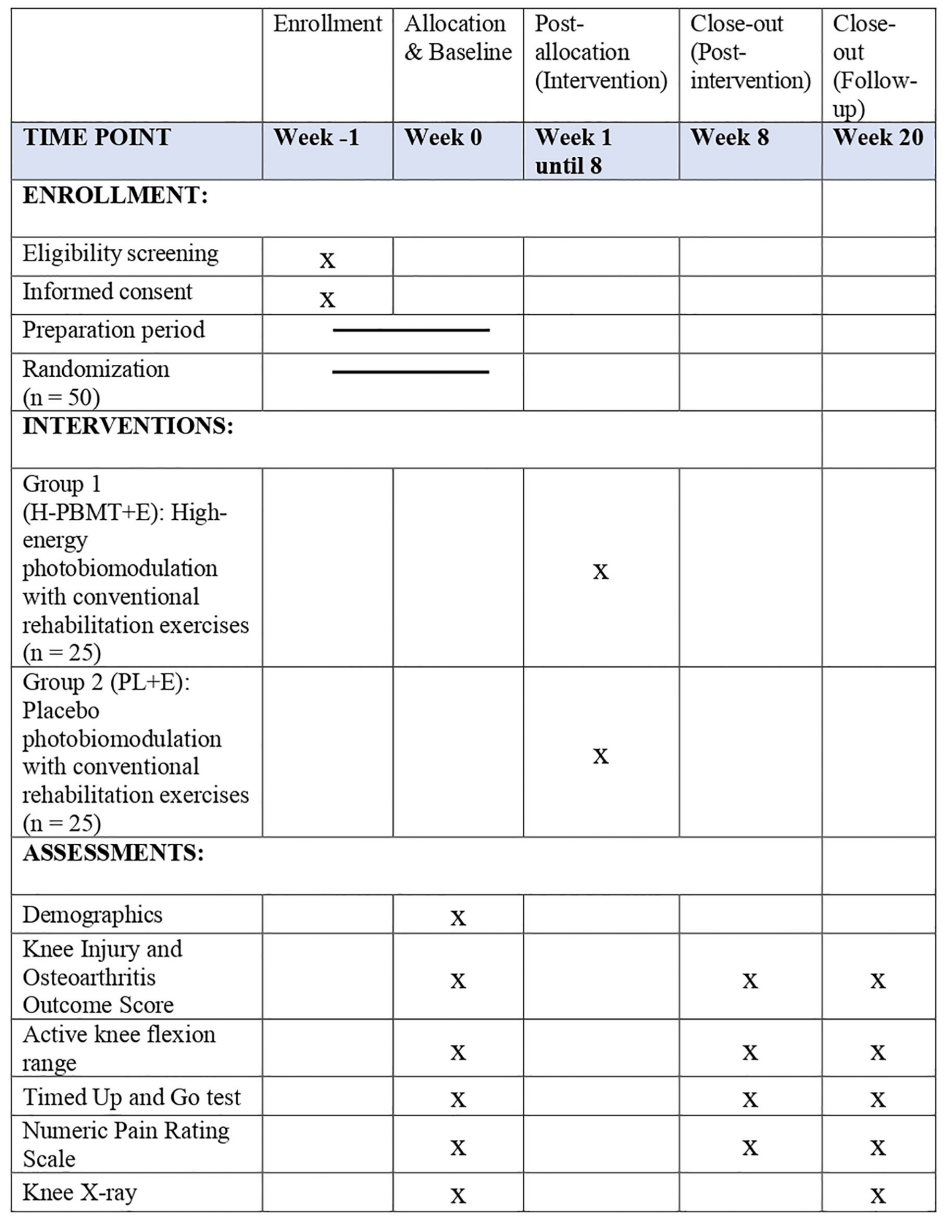
Schedule of enrolment, interventions, and assessments.

This study will be a parallel-group, randomized, double-blind, placebo-controlled trial. Both participants and outcome assessors will be blinded to the group allocations to minimize bias. The trial design adheres to the Consolidated Standards of Reporting Trials (CONSORT) guidelines and follows the Standard Protocol Items Recommendations for Interventional Trials (SPIRIT) checklist (S1 File), ensuring high standards of methodological rigor and transparency in reporting. The study flow outlines the planned schedule for participant enrollment, interventions, and assessments at designated time points throughout the trial as detailed in Fig 2.

### Ethical approval and trial registration

Ethical approval for this study was granted by the Research Ethics Committee of Universiti Kebangsaan Malaysia (REC UKM JEP-2024-541) on 24 July 2024, in accordance with the ethical principles outlined in the Declaration of Helsinki (S2 File). The study protocol has been prospectively registered in the Australian New Zealand Clinical Trials Registry (ACTRN12624000699561p), a registry accredited by the World Health Organization’s International Clinical Trials Registry Platform. Any subsequent amendments to the protocol will be reported to the ethics committee and updated in the registry, with clear justifications provided for each change.

### Study setting

This study will be conducted at the Physiotherapy Department of Hospital Canselor Tuanku Muhriz (HCTM), Universiti Kebangsaan Malaysia (UKM), Malaysia. HCTM is a leading tertiary referral hospital in Malaysia, known for handling a high volume of rehabilitation cases. Its well-established infrastructure and clinical expertise in musculoskeletal rehabilitation make it an ideal environment for conducting this research, ensuring access to a diverse patient population and comprehensive clinical support.

### Target population

The target population will consist of adults diagnosed with symptomatic KOA according to the American College of Rheumatology (ACR) clinical and radiographic criteria, with the diagnosis made by orthopaedic physicians from the Primary Clinics of HCTM UKM. The inclusion criteria are as follows: (i) adults aged 40–70 of both genders, (ii) diagnosis of unilateral or bilateral symptomatic KOA based on the ACR criteria, defined as the presence of knee pain and meeting any three of the following criteria: age of 50 years or older, morning stiffness lasting less than 30 minutes, crepitus (grating sensation) on active motion, bony tenderness, bony enlargement, and absence of palpable warmth in the knee joint, (iii) confirmation of mild or moderate KOA severity with a Kellgren-Lawrence classification based on radiographic evidence from a knee radiograph conducted within the last six months, (iv) symptomatic knee pain with a minimum score of 3 out of 10 on the visual analogue scale [22], and (v) the ability to participate in the intervention and assessment program without restrictions. For participants with KOA in both knees, the knee with more severe symptoms, as indicated by the pain score, will be included. If both knees exhibit identical symptoms, the dominant knee will be assessed.

The exclusion criteria include: (i) the presence of other pathological conditions such as rheumatic diseases, prior hip or knee joint replacement, congenital dysplasia, osteochondritis dissecans, intra-articular fractures, septic arthritis, or acute ligament or meniscus injury, (ii) current participation in another KOA-related interventional study, (iii) use of prescription glucosamine sulfate, which could potentially affect the study’s outcomes, and (iv) having undergone intra-articular knee injections (e.g., corticosteroid, hyaluronic acid, or blood-derived products) for KOA management within the last six months.

### Sample size calculation

Sample size was calculated using G*Power software version 3.1.9.7. The calculation was based on the primary outcome, the Knee Injury and Osteoarthritis Outcome Score (KOOS), a validated, reliable tool commonly used to assess pain, symptoms, activities of daily living, sports/recreation, and quality of life in individuals with KOA [23]. The KOOS was selected as it provides a comprehensive assessment of both short-term symptomatic relief and long-term functional improvements, making it highly relevant for evaluating interventions like HILT in KOA patients. The calculation aimed to detect a minimal clinically important difference (MCID) of 11 points in the KOOS, ensuring that even modest but clinically significant changes could be captured [23].

The study aims to compare outcomes between two groups at three time points: baseline (week 0), immediately post-intervention (week 8), and three months follow-up (week 20). The calculation parameters were: (i) a pre-specified statistical power of 80%, equating to a beta level of 0.20, allowing for an 80% probability of detecting a true effect and a 20% risk of a type II error, (ii) an effect size of 0.2, derived from the MCID for KOOS, (iii) an alpha level of 0.05, signifying a 5% risk of a type I error, and (iv) an anticipated dropout rate of 25% [24]. Using these inputs, the minimum required sample size was estimated to be 50 participants, with 25 allocated to each group. In addition to the theoretical calculation, the selected sample size of 50 participants is consistent with several high-quality indexed studies that have examined the efficacy of PBMT and rehabilitation exercises in patients with KOA. Notably, this sample size aligns with findings from studies on H-PBMT in KOA populations, such as Ahmad et al. (2023) and Alayat et al. (2017), which included 34 to 45 participants and reported significant changes in outcomes [25, 26]. Moreover, a systematic review by Song et al. (2020) indicates that several randomized controlled trials in this area typically report sample sizes ranging from 30 to 50 participants, further supporting the feasibility of this chosen sample size [17].

### Procedures

**Fig 3:**
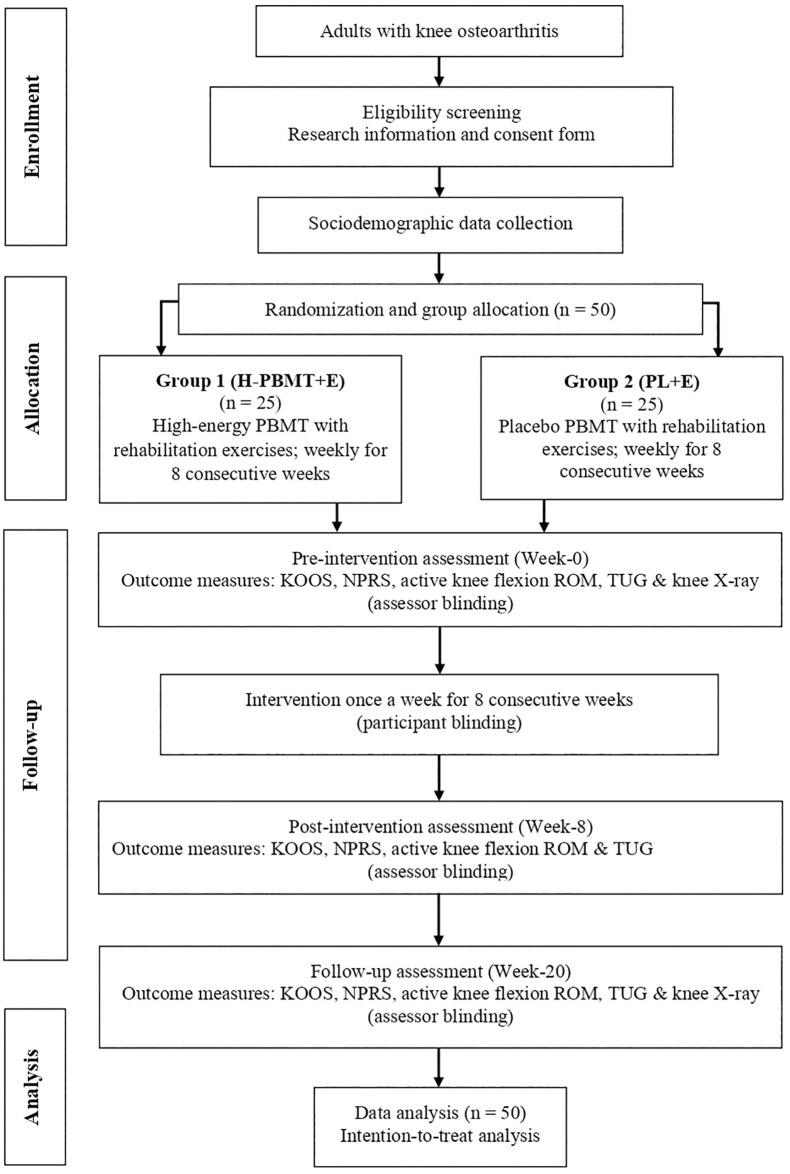
Study flowchart.

Patients with mild-to-moderate KOA will be screened and recruited from the Physiotherapy Department of HCTM using simple random sampling, provided they meet the predefined inclusion and exclusion criteria. Recruitment will be facilitated through various channels, including social media, online forums, referral programs, and by distributing brochures and posters at primary clinics within HCTM. Eligible participants will receive comprehensive verbal and written information about the study protocol and will be required to sign informed consent forms before enrollment. Sociodemographic data will be collected, and each participant will be assigned a unique identification number to ensure confidentiality. A total of 50 participants will be recruited and randomly allocated in a 1:1 ratio to one of two intervention groups using a computer-generated randomization table. Group 1 (H-PBMT+E; n = 25) will receive active high-energy photobiomodulation therapy for approximately 15 minutes, in addition to 45 minutes of conventional rehabilitation exercises, once a week for eight consecutive weeks. Group 2 (PL+E; n = 25) will receive a placebo photobiomodulation intervention for 15 minutes, alongside 45 minutes of conventional rehabilitation exercises, once a week for eight consecutive weeks. Allocation concealment will be ensured using sealed opaque envelopes containing the treatment group information. Screening, recruitment, and randomization will be conducted by a researcher who will not be involved in the intervention or outcome assessment. Fig 3 provides an overview of the study flowchart.

### Photobiomodulation intervention

**Fig 4:**
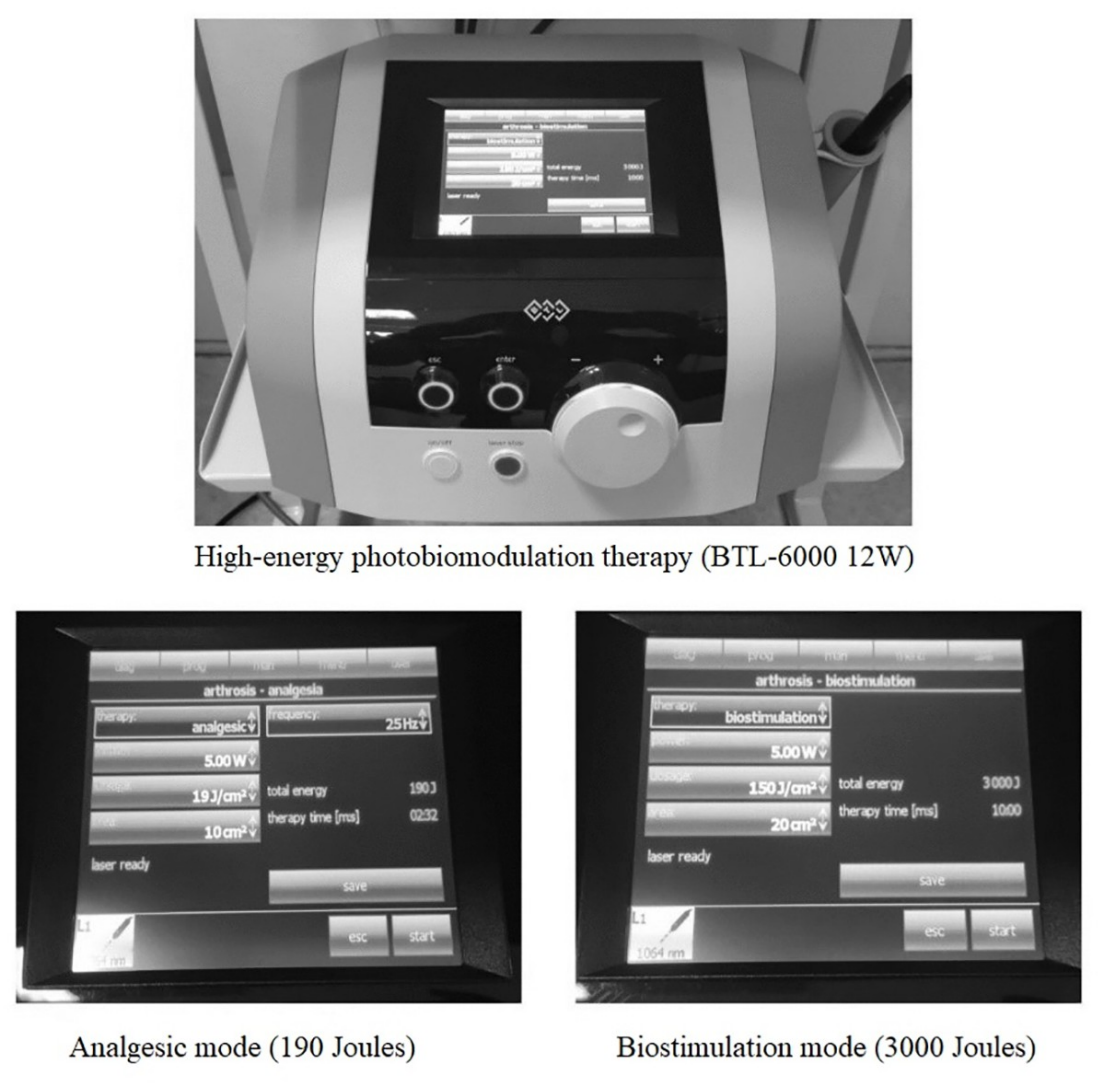
Analgesic and biostimulatory modes of photobiomodulation therapy.

In this trial, H-PBMT will be administered using the BTL-6000 High-Intensity Laser, operating at a wavelength of 1064 nm. The treatment protocol incorporates two distinct modes: (i) pulse mode to induce analgesic effects by modulating pain pathways and reducing nerve excitability, and (ii) continuous mode to promote tissue regeneration and enhance biostimulation. The pulse mode will be set to a frequency of 25 Hz, with a peak power of 5 W, a duty cycle of 25%, and an energy density of 19 J/cm^2^, applied over a 10 cm^2^ area for 5 minutes, delivering a total energy of 190 J per session [26, 27]. In the continuous mode, biostimulation will be delivered at a power output of 5 W, with an energy density of 150 J/cm^2^ applied over a 20 cm^2^ area for 10 minutes, resulting in a total energy delivery of 3000 J per session [26, 27]. The total energy delivered per session across both modes will be 3190 J, with each treatment session lasting 15 minutes (5 minutes of pulse mode and 10 minutes of continuous mode) [26] (Fig 4). The combination of these two modes offers dual benefits: immediate pain relief through pulse mode and longer-term tissue recovery through continuous mode [15].

**Fig 5:**
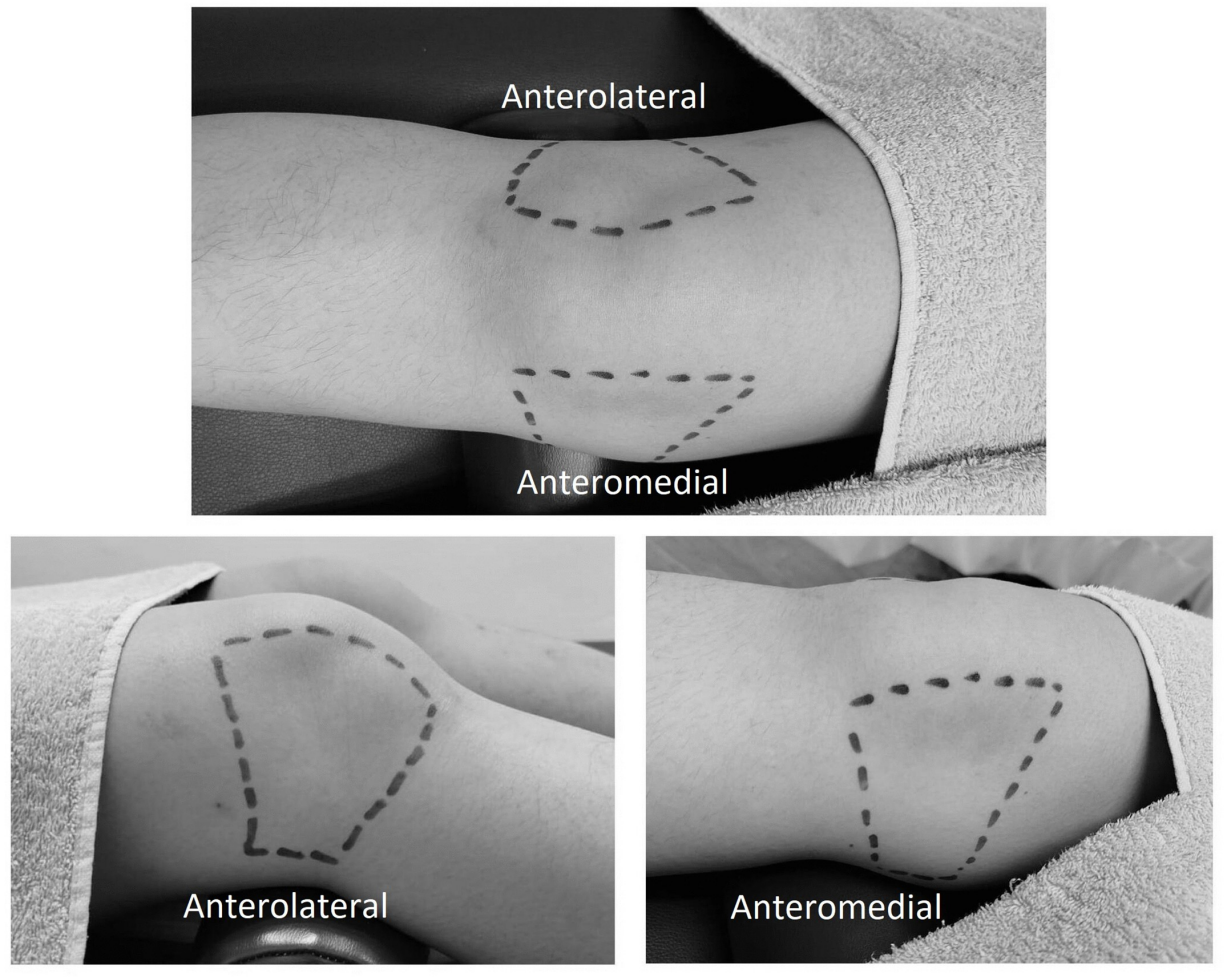
Treatment areas on the anteromedial and anterolateral knee.

Participants will be positioned in a supine position with their knee flexed at 30°, optimizing joint surface exposure to the laser [26, 27]. The treatment areas will target the antero-medial and antero-lateral regions of the knee joint [26, 27] (Fig 5). Based on the BTL manufacturer’s guidelines, the laser probe will be placed in vertical contact with the knee and moved slowly in a scanning motion, both longitudinally and perpendicularly [28]. This method is believed to ensure consistent energy delivery across the treatment area and helps avoid under-treatment of specific regions [28]. Safety precautions will be strictly enforced, with participants and therapists wearing protective laser eyewear throughout the procedure [28]. Participants will also be closely monitored for any sensations of discomfort or overheating to prevent superficial burns. To ensure consistency, the laser intervention will be administered by a single therapist for both groups, standardizing the application technique across all treatments. Participants in both the active photobiomodulation (H-PBMT+E) and placebo (PL+E) groups will undergo identical procedures, including visual light and acoustic indicators during the intervention to maintain blinding [29]. The placebo group will follow the same preparation and application process, except no energy will be emitted from the placebo device [29].

### Rehabilitation exercise

Qualified physiotherapists from HCTM UKM, who are blinded to the participants’ treatment allocation, will administer individualized physiotherapy treatment based on recommended KOA treatment guidelines for both groups. The rehabilitation exercises are grounded in evidence from prior studies demonstrating their efficacy in managing KOA [9, 30, 31], as well as current, validated guidelines for KOA management [7, 8, 32]. Specifically, the program follows protocols established by the Osteoarthritis Research Society International (OARSI) and draws from validated exercise regimens documented in the literature [8, 32], targeting key muscle groups integral to knee stability and function, such as the quadriceps, hamstrings, hip abductors, and calf muscles. The rehabilitation protocol is structured around progressive resistance exercises and functional tasks that address muscle weakness, joint instability, and reduced mobility, which are common impairments in KOA patients. Exercises include squats, sit to stand drills, step ups, calf raises, and both static and dynamic balance activities, with progression tailored to each participant’s tolerance and improvement to ensure optimal therapeutic efficacy [9, 33]. In addition, the current evidence-based practice for KOA rehabilitation recommends including patient education as a core component of treatment. In this study, appropriate patient education will be provided based on a structured rehabilitation package derived from previous relevant studies [31, 34], ensuring participants receive comprehensive guidance on KOA management.

The exercise program will include the following components:

1. Range of Motion Exercises: Prone knee bends (2 sets of 10 repetitions) and supine alternate knee bends (2 sets of 10 repetitions).
2. Stretching Exercises: Standing quadriceps stretch (1 set of 3–5 repetitions, holding each for 15 seconds), calf stretch in long sitting (1 set of 3–5 repetitions, holding each for 15 seconds), and supine hamstring stretch (1 set of 3–5 repetitions, holding each for 15 seconds).
3. Strengthening Exercises: Progression for sitting knee extensions, supine straight leg raises, static quadriceps exercises, and side-lying straight includes:
  - Week 1–2: 1 set of 5 repetitions with a 5-second hold
  - Week 3–5: 1 set of 7–10 repetitions with a 5–10 second hold
  - Week 6–8: 2 sets of 10 repetitions with a 10-second hold
4. Functional Training: Sit-to-stand exercises (10 repetitions), standing mini squats (10 repetitions), and walking exercises (approximately 2 minutes per session).

Each session will last approximately one hour and will be conducted once a week for eight weeks, with adjustments based on the patient’s assessment and clinical progression. The exercises provided serve as general guidelines; however, they will be customized according to individual assessments to accommodate each participant’s physical condition, progress, and response to treatment [9, 33]. To optimize adherence and effectiveness, participants will receive personalized feedback and regular follow ups, with monitoring including attendance tracking, session logs, and periodic evaluations to ensure compliance with the prescribed regimen [31].

### Outcome measures

The outcome measures will include: (i) self-report measures such as the KOOS and the Numerical Pain Rating Scale (NPRS), (ii) clinician-reported measures such as knee flexion active range of motion (AROM), (iii) performance-based measures such as the Timed Up and Go (TUG) test, and (iv) radiographic imaging (X-ray), providing a comprehensive evaluation of the health and functioning of the KOA population [35]. Outcome measures will be assessed at three key intervals: baseline (pre-intervention, week 0), immediately post-intervention (week 8), and follow-up at three months after the final treatment session (week 20). All assessments will be conducted by a blinded assessor using standardized tools and protocols.

The primary outcome measure of this study is the KOOS, which evaluates five domains: pain, symptoms, activities of daily living, sports/recreation, and quality of life. KOOS was selected due to its sensitivity in detecting clinically meaningful changes across a broad spectrum of knee function, aligning with the goal to assess both symptomatic and functional outcomes in KOA patients [23, 36]. Additionally, KOOS is widely used in both clinical and research settings for knee-related conditions, providing a robust framework for comparison with other studies [36]. The KOOS demonstrates high reliability and validity across different languages, with excellent test-retest reliability, as evidenced by intraclass correlation coefficients (ICCs) ranging from 0.91 to 0.99 [36]. The NPRS is an 11-point scale ranging from 0 to 10, used to assess pain intensity, where 0 indicates "no pain" and 10 represents "the worst imaginable pain." Participants will select the number that best represents their current level of pain. The NPRS has demonstrated excellent reliability, with an ICC of 0.95 [37].

Knee flexion will be measured using a goniometer, a tool known for its high reliability in assessing knee ROM. The ICC for knee flexion measurement is reported at 0.997 [38]. The goniometer provides consistent results across various positions, including supine, prone, and sitting, with high correlation coefficients (r > 0.80) [38]. The TUG test measures functional mobility by timing the duration required to stand from a seated position, walk 3 meters, and return to the seated position. It is particularly useful for assessing functional mobility in individuals with mild to moderate grade of KOA [39]. The test has demonstrated high reliability and validity, with a minimal detectable change of 1.10 seconds [39]. Recommended by the Osteoarthritis Research Society International for KOA patients, the TUG test shows strong intra-rater and inter-rater reliability, with ICC values of 0.97 and 0.96, respectively [39].

Knee radiographic imaging will be conducted at baseline and during the 3-month follow-up interval to quantitatively assess changes in knee joint structure. A qualified radiologist will perform the X-ray examinations to ensure standardized assessments and minimize assessor bias [40]. The evaluation will focus on key parameters, including joint space width (to assess joint space narrowing), joint alignment (to evaluate angles such as the mechanical axis of the leg for varus or valgus deformities), and cartilage thickness (to measure articular cartilage thickness in specific knee joint regions to monitor cartilage loss). The use of X-rays as an outcome measure is intended to evaluate the severity and progression of KOA by examining changes in joint space, the presence of osteophytes, and the degree of bone or joint destruction [4, 40]. X-rays are a practical choice due to their relatively low cost and widespread availability in clinical and research settings [40]. Extensive research has confirmed the reliability and validity of using X-rays to assess KOA, showing high inter-observer reliability and moderate-to-high validity compared to other imaging modalities such as magnetic resonance imaging or diagnostic ultrasound [40].

### Blinding

This study will employ a rigorous double-blinded design to minimize bias and ensure the validity and reliability of the trial outcomes [41]. Both participants and outcome assessors will be unaware of group allocations to prevent expectation or observer bias from influencing the results. The treatment devices will be identical in appearance and handling, with the placebo device emitting light without therapeutic radiation, thus replicating the visual and procedural characteristics of the active H-PBMT treatment [41]. Additionally, all participants will wear standardized protective eyewear, further concealing the nature of the treatment. Outcome assessors will not participate in the intervention and will be kept separate from information about group allocations. To prevent any potential unintentional disclosure of treatment information, assessors will be trained to limit their interactions with participants, ensuring that evaluations take place in a location entirely distinct from the intervention area [41].

### Evaluation of blinding success

The effectiveness of blinding will be assessed using a brief blinding questionnaire administered to both participants and assessors at the study’s conclusion [42]. This questionnaire will ask participants, "Which treatment do you believe you received (the choices being H-PBMT+E or PL+E)?" and assessors, "Which treatment group do you think this participant was assigned to (H-PBMT+E or PL+E)?" The responses will be analyzed to determine the accuracy of the guesses. Additionally, the Bang Blinding Index (BBI) will be used to measure blinding success, where a BBI of 0 indicates perfect blinding, positive values reflect correct treatment guesses, and negative values suggest placebo guesses occurring more frequently than would be expected by chance [42]. The results from the questionnaires and BBI will be analyzed to evaluate the effectiveness of blinding. Any significant deviations from 0 or signs of unblinding will be carefully investigated to understand their causes and implications [42]. Findings regarding blinding success, as well as any challenges or breaches, will be documented and discussed in the final manuscript to assess their potential impact on study outcomes. This comprehensive approach to blinding aims to minimize bias and enhance the validity and reliability of the trial [42]. Moreover, continuous monitoring of the blinding process will be conducted throughout the trial, with interim checks to detect any potential breaches early on. These rigorous measures are designed to uphold the highest standard of scientific integrity throughout the study.

### Safety consideration

H-PBMT is generally considered safe when administered within established guidelines, but certain precautions are necessary due to its high-intensity nature [14]. The primary safety concern is the risk of ocular damage, requiring mandatory protective eyewear for both patients and practitioners during all sessions [28, 43]. Without proper protection, the emitted energy levels could cause retinal injury [28, 43]. In this trial, participants will be informed about potential mild side effects, such as redness, localized swelling, or discomfort at the treatment site [14]. These reactions are usually transient, but participants will be closely monitored, and any severe adverse events will be addressed immediately. A thorough review of each participant’s medical history will identify contraindications, including photosensitivity, active malignancies, or implanted medical devices [28]. Interim analyses will be conducted at predefined intervals to evaluate both safety and efficacy. Adverse events will be tracked, and in the event of significant safety concerns, the research team will review the data and decide whether to modify or discontinue the treatment, ensuring participant safety while maintaining the integrity of the trial.

### Data monitoring, access and dissemination policy

A data monitoring committee will not be established for this trial due to the non-invasive nature of the protocol, which poses minimal risk to participants. Instead, regular monitoring by the research team will be implemented to ensure participant safety and adherence to the trial procedures. The trial is fully independent of the sponsor, with no involvement from the funder in the design, conduct, analysis, or interpretation of the results. Access to the final trial dataset will be restricted to the principal investigator and authorized members of the research team. No contractual agreements limit the investigators’ access to the dataset, and the sponsor and funder will not have access to the raw data. This ensures the research team’s full autonomy in data handling, analysis, and reporting. The complete trial protocol is publicly available through the ANZCTR registry, and the raw dataset can be provided upon reasonable request, pending approval from the principal investigator.

### Statistical analysis

The data for this study will be analysed using SPSS version 27.0, adhering to the intention-to-treat principle, which includes all participants regardless of their adherence to the intervention. Data entry, security, and storage will follow strict protocols to ensure quality and confidentiality. Double data entry will be used to reduce errors, and range checks will catch any outliers or inconsistencies. All data will be encrypted and stored in a password-protected system with limited access, complying with privacy regulations. Missing data will be handled using the multiple imputation technique, which involves creating multiple datasets by imputing missing values through statistical models [44]. The results from these datasets will then be combined to account for the uncertainty caused by the missing data. Multiple imputation is considered a more suitable method for addressing missing data in clinical studies compared to the last observation carried forward method, which may introduce bias by retaining the last observed value [44]. In contrast, multiple imputation provides a more robust approach by appropriately managing the uncertainty related to missing data [44]. Sensitivity analyses will also be conducted to assess the robustness of different methods for handling missing data. Sociodemographic characteristics of participants at baseline will be described using descriptive statistics and cross-tabulations. Baseline comparability analysis of the clinical outcomes between groups will be performed.

The primary statistical analysis will employ repeated measures ANOVA to assess outcomes, provided the assumptions of normality and sphericity are met. Normality will be evaluated using the Shapiro-Wilk test, with higher values indicating more normal data, while sphericity will be assessed using Mauchly’s test, where lower values closer to 0 suggest more homogenous variance between levels [24]. A two-way repeated measure (2 x 3) ANOVA will be conducted to examine the effects of time, group, and their interaction. Effect sizes will be determined using Cohen’s d, with values of 0.2, 0.5, and 0.8 indicating small, medium, and large effects, respectively [24]. To address the risk of multiplicity due to multiple endpoints, Bonferroni correction will be applied. This adjustment will lower the significance threshold based on the number of comparisons to mitigate the risk of Type I errors (false positives) from multiple testing. During the baseline to post-intervention (week 8) assessment interval, the primary outcome will be the KOOS, with secondary outcomes including VAS, TUG, and active knee flexion ROM. For the baseline to 3-month follow-up (week 20), radiographic structural changes assessed via X-ray will be the primary outcomes, given the need for extended periods to observe structural alterations.

Additional sensitivity analyses will be conducted based on KOA severity (mild vs. moderate) and age groups (younger vs. older adults). Clinically meaningful changes will be evaluated using the minimal clinically important difference (MCID) for measures such as KOOS, NPRS, active knee flexion range, and the TUG test to enhance the interpretability of clinical relevance [23]. An alpha level of 0.05 will be maintained for all statistical tests.

## Results

Participant screening and recruitment are scheduled to commence in October 2024, with the completion of data collection anticipated by October 2025. The findings from this trial will be disseminated through peer-reviewed publications, conference presentations, and data-sharing platforms. A detailed data policy statement outlining data access and sharing procedures will be made available, ensuring transparency and compliance with ethical standards for data sharing within the scientific community. Any amendments to the study protocol will be promptly notified to the ethics committee and the trial registry, ensuring full compliance with ethical and regulatory requirements.

## Discussion

This study aims to deepen the understanding of H-PBMT in combination with therapeutic exercise for KOA by examining its short-term and sustained effects on knee pain, physical function, and radiographic morphological changes. Unlike prior studies, which have primarily focused on H-PBMT’s role in symptomatic relief [13, 16, 17], this study will explore its potential to modify disease progression, specifically through radiographic assessments. By investigating these structural effects, we aim to address an essential gap in the literature.

An important aspect of this study is its focus on participants aged 40 to 70 years, aligning with evidence indicating that KOA prevalence increases significantly after age 40 and peaks between ages 50 and 70 [5, 45]. By concentrating on participants within this age range, the study aims to improve the relevance and applicability of the findings for those most likely to benefit from therapeutic interventions, such as H-PBMT and rehabilitation exercises. In addition, the integration of H-PBMT with conventional rehabilitation exercises is hypothesized to provide synergistic benefits, potentially yielding both symptomatic and structural improvements for KOA patients. The study’s robust design, incorporating a placebo controlled, double blinded methodology, will ensure that observed effects are directly attributable to the intervention, reducing the likelihood of placebo or expectancy biases [42]. By employing various outcome measures, such as the KOOS, TUG test, and knee X rays, this study will comprehensively evaluate the intervention’s efficacy [35].

Radiographic changes in KOA, such as joint space narrowing and osteophyte formation, are critical indicators of disease progression and severity [40, 46]. These changes offer objective evidence of the structural impact of the disease, essential for evaluating the sustained effectiveness of therapeutic interventions over time [46]. Although our study involves a 3-month follow-up, assessing these outcomes is crucial for understanding the longer-term benefits of the treatment strategy beyond immediate symptom relief [21]. This evaluation will help determine whether interventions like H-PBMT not only alleviate symptoms but also slow or halt radiographic progression, contributing to long-term joint health and functionality. The non-invasive nature and tolerability of H-PBMT further enhance its appeal as a viable intervention for individuals with KOA [43].

This study’s novel focus on assessing structural changes in KOA through H-PBMT warrants a deeper exploration of the potential implications of laser intensity and dose parameters on cartilage integrity. Evidence from animal models suggests an intensity-dependent response; for instance, Xiang et al. (2020) conducted a meta-analysis demonstrating that lower irradiances (<1 W/cm^2^) were associated with significant improvements in cartilage structural integrity and chondrocyte distribution, emphasizing the importance of specific laser parameters for optimal therapeutic outcomes in cartilage repair [47]. These findings suggest that carefully controlled laser intensity may enhance chondroprotective effects, promoting cartilage regeneration in KOA models [47]. In another animal study, Balbinot et al. (2021) investigated dosimetric challenges in LED-based PBMT for KOA, demonstrating that light dose reaching the articular cartilage varies significantly based on light source characteristics, such as divergence angle, wavelength, and irradiation location [19]. Their findings underscore the need for precise parameterization in PBMT to achieve effective cartilage penetration, with positioning, particularly around the patella, shown to maximize dose delivery to the cartilage, potentially enhancing therapeutic outcomes [19]. Nevertheless, clinical studies assessing the structural effects of PBMT on cartilage in humans are limited, especially regarding optimal dose distribution [48]. This highlights a significant gap, as structural outcomes are crucial for understanding the potential for sustained disease modification in KOA [48]. Given these insights, the current study holds considerable clinical relevance by including the evaluation of structural outcomes in KOA, which may help determine if H-PBMT protocols observed in animal models yield comparable cartilage remodeling effects in humans. These findings could guide clinicians in optimizing laser parameters, including dose and positioning, to enhance the therapeutic efficacy of PBMT in managing KOA.

However, this study has certain limitations that warrant acknowledgment and mitigation efforts. First, although participant blinding will be maintained by using identical visual light and acoustic signals in placebo treatments, H-PBMT can slightly increase skin temperature during active treatments, as reported by Joensen et al. (2011) [49]. This mild thermal effect introduces a small risk that participants in the experimental group might detect the active intervention, potentially compromising the blinding process [49]. To address this, the success of blinding will be assessed using a blinding questionnaire and the Bang Blinding Index, with results reported to evaluate and mitigate any bias. Secondly, while the 3-month follow-up period is expected to provide insights into the intervention’s sustained effects, it may not fully capture the long-term disease-modifying potential of H-PBMT on KOA. It is recognized that clinical trials with longer follow-up periods often encounter challenges with adherence and higher dropout rates [9, 20]. Since adherence to both the intervention and rehabilitation program is essential, we have incorporated strategies to promote adherence, including regular reminders and session logs. Despite these measures, non-adherence may still impact the outcomes, particularly if it leads to differential adherence between groups or missing data. To mitigate this, data will be analyzed according to the intention-to-treat principle to preserve the benefits of randomization and enhance the reliability of findings [44]. Lastly, while radiographic imaging is valuable for assessing structural changes, it cannot directly visualize cartilage [40]. Considering these limitations, future research is encouraged to adopt extended follow-up intervals to thoroughly assess the intervention’s long-term efficacy on symptom relief and structural preservation. Additionally, incorporating advanced imaging techniques, such as MRI, could enhance the ability to assess joint structural changes in greater detail [40].

## Conclusion

In conclusion, this study aims to fill a critical research gap by evaluating the disease-modifying potential of H-PBMT combined with therapeutic exercise in patients with KOA. The findings will not only inform clinical practice but also potentially lead to the development of enhanced treatment protocols that offer both symptomatic relief and structural benefits. By bridging this knowledge gap, the research could contribute to more effective, evidence-based management strategies and improved outcomes for individuals suffering from KOA.

## Supporting information

S1 File SPIRIT checklist. (PDF)

S2 File Ethical approval letter. (PDF)
