# Supplementary material for: Effects of photobiomodulation combined with rehabilitation exercise on pain, physical function, and radiographic changes in mild to moderate knee osteoarthritis: A randomized controlled trial protocol
Source: PLoS One. 2025 Jan 21;20(1):e0314869. doi: 10.1371/journal.pone.0314869 (PMC11750081; doi:10.1371/journal.pone.0314869)
Supplement: S2 File — (PDF) [file pone.0314869.s002.pdf]

TITLE:

PHOTOBIOMODULATION AS ADJUNCTIVE TO REHABILITATION EXERCISE IN KNEE OSTEOARTHRITIS: A MIXED METHOD RESEARCH DESIGN

NAME OF RESEARCHERS:

PRINCIPAL INVESTIGATOR

DR MOHD AZZUAN BIN AHMAD (K020743)

PHYSIOTEHRAPY PROGRAM, CENTRE FOR REHABILITATION AND SPECIAL NEEDS STUDIES, FACULTY OF HEALTH SCIENCE, UKM

CO-INVESTIGATOR(S)

MISS TAY YAN LING

PROF. DR. DEVINDER KAUR A/P AJIT SINGH

PROF. MADYA DR. NOR HAMDAN BIN MOHAMAD YAHAYA

RESEARCH DURATION:

11 JULY 2024 – 10 JULY 2026 (24 MONTHS)

FACULTY OF HEALTH SCIENCES

UNIVERSITI KEBANGSAAN MALAYSIA

KUALA LUMPUR

Executive Summary

Knee osteoarthritis (KOA) is a prevalent chronic condition globally, contributing to knee pain and disability. Recent studies suggest that high-intensity laser therapy (HILT) holds promise as a treatment for KOA. However, clinical evidence supporting its efficacy, particularly over the long term, remains insufficient. This study aims to (i) assess the short- and long-term effects of HILT as a supplement to rehabilitative activities for individuals with mild to moderate KOA, focusing on joint morphologic changes, and (ii) explore the experiences and perceptions of individuals undergoing this combined intervention. The study will employ both quantitative and qualitative methodologies. The first phase of the study will be a randomized double-blinded controlled trial, which will involve 40 participants with mild-to-moderate KOA based on the Kellgren-Lawrence classification. Participants will be randomly assigned to either the HILT with exercise (HILT+E; n=20) or the placebo laser with exercise group (PL+E; n=20). For eight consecutive weeks, both groups will receive their prescribed laser treatment—HILT (5 W, 1064 nm, 19 to 150 J/cm2, and 3190 J each session) or a placebo—along with KOA rehabilitation exercises once a week. Examples of KOA rehabilitation exercises are range of motion, stretching, strengthening, and functional training exercises. The exercise prescription will be modified based on the individual's progression. The outcome measures included are knee X-ray (cartilage thickness and joint space), Knee Injury and Osteoarthritis Outcome Score (KOOS), Timed Up and Go (TUG) test, and Numeric Pain Rating Scale (NPRS), which will be evaluated at three different time points by a blinded assessor: baseline, post-intervention, and follow-up after three months of the last session. Knee X-rays (to evaluate cartilage thickness and joint space) will be taken at baseline and follow-up. Two-way repeated measures ANOVA will be used to assess the effects of time, group, and the interaction between the two groups. In the second phase of the study, structured one-to-one interviews will be carried out with participants to explore patient experiences. Each interview will be audio or video-recorded with participants' consent, and questions will be posed in a conversational manner to capture both verbal responses and non-verbal cues. The qualitative data will undergo thematic analysis using ATLAS.ti software programs. The findings of this study are pivotal for deepening our understanding of the impacts of HILT and establishing an effective treatment regimen for KOA. These insights have the potential to improve the pathological condition, providing optimism for mitigating KOA progression.

Keywords: Knee Osteoarthritis; Early stage of Knee Osteoarthritis; Exercise; High Intensity Laser Therapy; Laser therapy; long term efficacy; disease-modifying potential; Qualitative and quantitative approaches.

Table of Contents

1.1 Study background 6

1.2 Problem Statement 7

1.3 Research Questions 8

1.4 Research Objectives 8

1.4.1 Main Objectives 8

1.4.2 Specific Objectives 8

1.5 Research Hypothesis 9

1.6 Research Significance 9

1.7 Research Framework 11

2.0 Literature Review 12

2.1 Pathophysiology of KOA 12

2.2 Prevalence and Impact of KOA 12

2.3 Current Management of KOA 13

2.4 Mechanism of HILT 14

2.5 Clinical Evidence of HILT in KOA 15

2.6 Summary of Literature Findings 20

3.0 Methodology 21

3.1 Phase 1: Quantitative Design-Randomized Controlled Trial (Short-term Outcomes) 21

3.1.1 Study Design 21

3.1.2 Study Settings 21

3.1.3 Target Population 21

3.1.4 Sample Size Calculation 22

3.1.5 Sampling and randomization 22

3.1.6 Group allocation and intervention 23

3.1.7 Outcome Measures 25

3.1.8 Data Analysis 26

3.2 Phase 2: Qualitative Study-Structured One-On-One Interviews (Participants' Psychological Well-being, Experiences, and Perspectives) 27

3.2.1 Procedures 27

3.2.2 Sampling method 27

3.2.3 Outcomes Measures 28

3.2.4 Data Analysis 29

3.3.3 Ethic consideration and participation confidentially 29

3.3.4 Budget 29
3.3.5 Flow Chart of Trial 32
4.0 Reference list 35

1.0 Introduction

1.1 Study background

Knee osteoarthritis (OA) is a common cause of knee pain worldwide, which is caused by repetitive stress and mechanical trauma leading to an inflammatory process within the knee joint, resulting in articular cartilage deterioration and loss of chondrocytes (Geng, Li et al. 2023). The primary method of dealing with musculoskeletal pain involves conservative techniques that include both pharmaceutical and nonpharmacological therapies. Recent research has demonstrated the effectiveness of nonpharmacological interventions, including exercise, heat modalities, and manual therapy (Soares Fonseca, Pereira Silva et al. 2023). Despite the guidelines for treating knee OA prescribe workouts on land or in the water, weight loss, and the use of a cane, alternatives are investigated for pain reduction, including topical NSAIDs, intraarticular corticosteroid injections, and physical modalities like laser therapy. High-intensity laser therapy (HILT), distinguished from low-level laser therapy (LLLT) by its greater output power, has shown positive effects on pain relief, range of motion, and functional improvement in knee OA (Siriratna, Ratanasutiranont et al. 2022). However, the lack of a standardized treatment protocol for HILT and significant variation in study outcomes highlight the need for further research in this area. This study aims to investigate the short-and long-term effects of high-intensity laser therapy combined with rehabilitation exercise on knee pain in adults with early knee osteoarthritis. The study also attempts to evaluate the long-term effects of high-intensity laser therapy combined with rehabilitation exercise on physical function and knee-related disability in adults with early knee osteoarthritis. Additionally, by combining high-intensity laser therapy with rehabilitation exercises, the study aims to investigate the potential disease-modifying effects of the treatment. Specifically, it focuses on how the treatment affects the structural aspects of the knee joint in adults with early-stage osteoarthritis. The study will be carried out using quantitative and qualitative approaches in a parallel-group, randomised, double-blind (subjects and outcomes assessor), placebo-controlled trial design. There will be three stages of it. Phase 1 involves a random allocation of 40 individuals to participate in one of two groups: 20 individuals in group 1 will be treated with high intensity laser treatment with exercise (HILT+E; n = 20), while another 20 participants in group 2 will receive exercise and a placebo laser (PL+E; n = 20). Each group is scheduled for weekly 60-minute sessions for eight weeks in consecutive weeks. However, phase 2 of the study extends an 12-week follow-up period to investigate the long-term effects of high intensity laser therapy in comparison to placebo laser therapy and exercise. All 40 participants from both groups are

included in this phase, which examines knee pain, physical function, and psychological health. Phase 3 on the other hand, emphasises the qualitative aspect and demonstrates the method to carry out structured one-on-one interviews with participants to discover more about their points of view, experiences, and feelings expressed. Phase 1 is significant due to the fact that it offers an in-depth examination of the combined intervention's short-term effects. It provides immediate data regarding how it affects physical function, psychological health, and knee pain. Phase 2 extends this inquiry, emphasising the long-term effects over a 12-weeks follow-up period, resulting in valuable data on sustained improvements. Phase 3 captures the experiences and perspectives of the patients, providing a deeper significance to the overall findings through qualitative investigation through structured interviews. Therefore, the combination of exercise and high intensity laser therapy, with an emphasis on holistic well-being and long-term effects, has the potential to reverse the damage caused by knee osteoarthritis. This will also provide a comprehensive comprehension of the effects of treatment and patient experiences, which will improve global management strategies.

1.2 Problem Statement

Knee osteoarthritis (KOA) is a prevalent chronic condition affecting numerous individuals and healthcare systems globally. Despite the availability of various treatments, the search for effective disease-modifying therapies for KOA continues. High-intensity laser therapy (HILT) has emerged as a promising intervention for alleviating knee pain, enhancing physical function, and reducing disability in adults with mild KOA (Wu, Luan et al. 2022, Ahmad, Moganan et al. 2023). However, the incorporation of HILT into mainstream KOA management is hindered by the absence of high-quality evidence supporting its efficacy, as highlighted by current treatment guidelines. Previous research on HILT in KOA has predominantly focused on short-term effects and often involved severe KOA cases, limiting the understanding of its long-term effectiveness, especially in managing a chronic condition like KOA.

Moreover, the existing body of research has primarily emphasized quantitative outcomes, neglecting a comprehensive exploration of qualitative aspects. There is a notable lack of studies delving into the qualitative dimensions of HILT in KOA, with a focus on patients' experiences, perceptions, and the impact of the therapy on their daily lives. This dearth of qualitative research poses a significant gap in understanding the holistic effects and patient-centered outcomes associated with HILT in KOA. Therefore, this study aims not only to assess

the quantitative effectiveness of HILT but also to address the lack of qualitative exploration, providing a more comprehensive understanding of its role as an adjunctive therapy to KOA rehabilitation exercises. By incorporating both quantitative and qualitative methodologies, the study strives to offer a more nuanced perspective on HILT's disease-modifying potential in ameliorating knee joint damage during the early stages of KOA. This comprehensive approach will contribute valuable insights to the existing knowledge base and may influence future guidelines for KOA management, ensuring a more holistic and patient-centered approach.

1.3 Research Questions

1. What are the short-and long-term effects of high-intensity laser therapy (HILT) combined with rehabilitation exercise on knee pain, physical function, and disability level among adults with early knee osteoarthritis (KOA)? Given the limited knowledge of HILT outcomes, especially in the context of its long-term effectiveness for early KOA.
2. What are the lived experiences and perceptions of individuals with early knee osteoarthritis who undergo a combined intervention of high-intensity laser therapy (HILT) and rehabilitation exercise, and how do these experiences influence their daily lives and well-being?

1.4 Research Objectives

1.4.1 Main Objectives

1) To examine the long-term effects of high-intensity laser therapy (HILT) combined with rehabilitation exercise on knee pain, physical function, and disability levels among adults with early knee osteoarthritis (KOA).
2) To investigate the lived experiences and perceptions of individuals with early knee osteoarthritis undergoing the combined intervention of HILT and rehabilitation exercise.

1.4.2 Specific Objectives

1) To examine the short-and long-term effects of HILT combined with rehabilitation exercise on knee pain, physical function and knee-related disability in adults with mild to moderate KOA.
2) To examine the potential disease-modifying effects of high-intensity laser therapy in combination with rehabilitation exercise in improving knee joint structure in adults with early knee osteoarthritis.

3) To evaluate patient-centered outcomes, including the impact on daily activities, quality of life, and overall patient satisfaction, to gain a more holistic perspective on the effectiveness of the combined intervention.

1.5 Research Hypothesis

1) Combining high-intensity laser therapy with rehabilitation exercise will significantly reduce knee pain, improve physical function, and reduce knee-related disability levels among adults with early knee osteoarthritis in the long term.
2) Combining high-intensity laser therapy with rehabilitation exercise will report positive lived experiences and perceptions. These experiences will significantly contribute to improved daily functioning, enhanced quality of life, and overall patient satisfaction compared to those in the control group.
3) Combining HILT with rehabilitation exercise will report positive lived experiences and perceptions. These experiences will significantly contribute to improved daily functioning, enhanced quality of life, and overall patient satisfaction compared to those in the control group.

1.6 Research Significance

The proposed study on the short-and long-term effects of high-intensity laser therapy (HILT) combined with rehabilitation exercise on knee pain in adults with early knee osteoarthritis (KOA) addresses a critical gap in the current research landscape. Knee osteoarthritis is a prevalent chronic condition, and despite various available treatments, the quest for effective disease-modifying therapies continues. The study recognizes the promising potential of HILT but acknowledges the hindrance in its incorporation into mainstream KOA management due to the absence of robust evidence supporting its efficacy, particularly in the context of long-term outcomes. One significant aspect of the research lies in its comprehensive approach, combining both quantitative and qualitative methodologies. While previous studies have primarily emphasized quantitative outcomes, this study recognizes the importance of delving into the qualitative dimensions of HILT in KOA. The inclusion of structured one-on-one interviews in the third phase aims to capture the lived experiences and perceptions of individuals undergoing the combined intervention of HILT and rehabilitation exercise. This qualitative investigation is crucial in understanding the holistic effects of the treatment and its impact on patients' daily lives and well-being.

Furthermore, the research objectives encompass a multifaceted examination of the intervention. The study aims not only to assess the short-and long-term effects on knee pain, physical function, and disability levels but also delves into the potential disease-modifying effects on knee joint structure. This holistic approach recognizes the complexity of KOA and aims to contribute valuable insights that can inform future guidelines for KOA management. The research questions and hypotheses highlight the study's focus on addressing the limited knowledge of HILT outcomes, especially its long-term effectiveness for early KOA. By investigating both the quantitative effectiveness and qualitative experiences, the study aims to provide a nuanced perspective on the disease-modifying potential of HILT in ameliorating knee joint damage. In conclusion, the significance of this research lies in its holistic and patient-centered approach to understanding the effectiveness of combining HILT with rehabilitation exercise for early knee osteoarthritis. The integration of qualitative exploration, emphasis on long-term outcomes, and consideration of disease-modifying effects contribute to advancing our understanding of KOA management strategies.

1.7 Research Framework

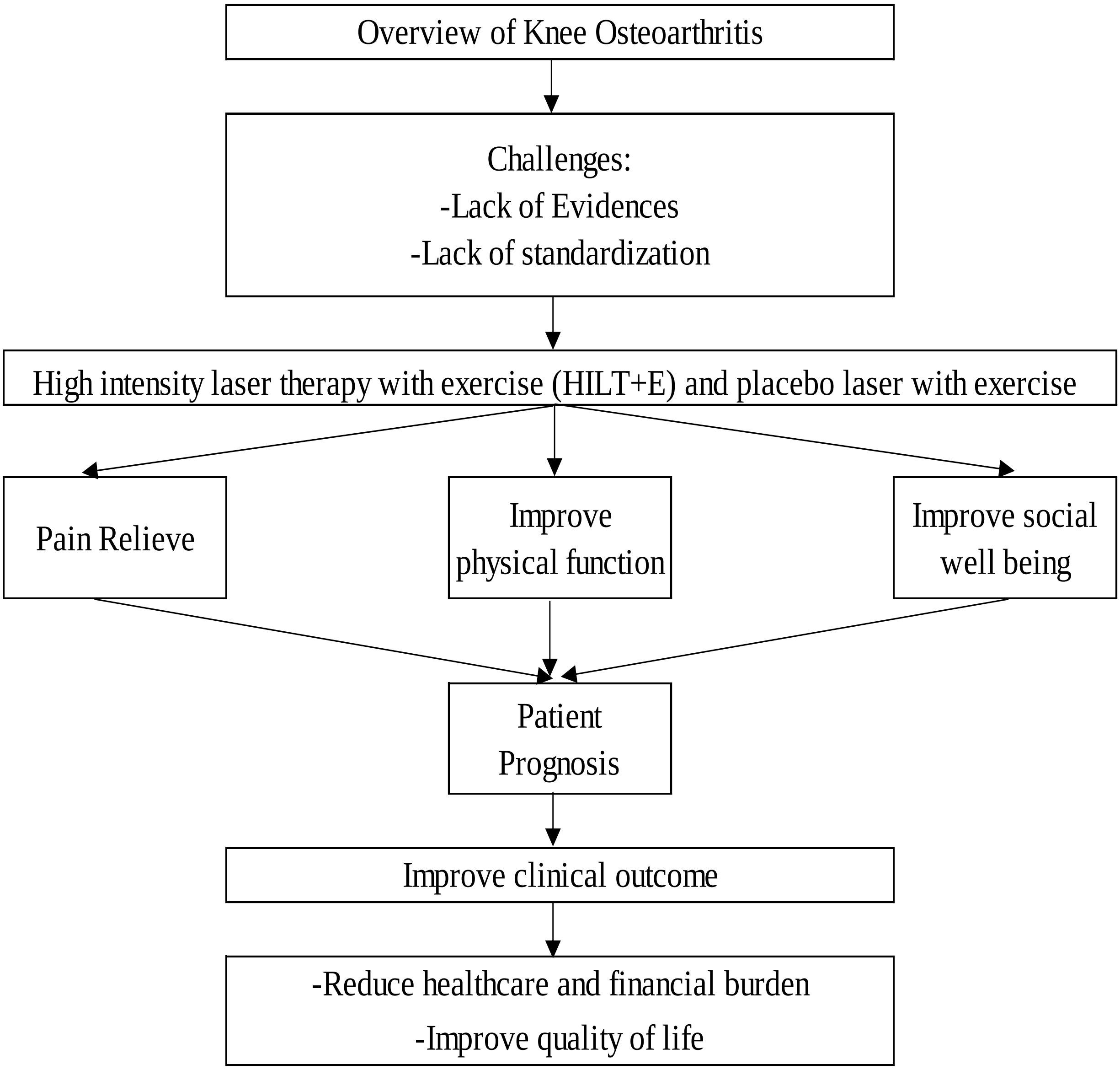

2.0 Literature Review

The literature review will explore the pathophysiology of knee osteoarthritis (KOA), its prevalence, and the resultant implications of KOA on both physical and psychological health. This review will comprehensively examine the mechanism, prevalence, and consequences of knee osteoarthritis (KOA) on physical and psychological health. A thorough investigation of current management strategies is expected to precede an in-depth study of laser therapy as an alternative treatment approach. The principles of laser therapy will be thoroughly investigated, and clinical evidence regarding the effects of the treatment on pain, physical function, and psychological health will be evaluated. The conclusion of the review will encompass key findings while emphasizing prevalent gaps in knowledge, thereby providing a basis for future studies.

2.1 Pathophysiology of KOA

Current clinical investigations are actively delving into the elusive origins of KOA, and recent findings suggest a lack of a definitive understanding (Lv, Yang et al. 2021). It is presently recognized as a type of panarthritis, impacting every aspect of a joint, including calcified cartilage, subchondral bone, capsular ligaments, and synovial fluid (Du, Liu et al. 2023). KOA may arise due to various factors, with mechanical factors such as engaging in repetitive activities (e.g., frequent kneeling, heavy lifting) and having weak knee extensor muscles identified as crucial contributors to its prevalence, rendering joints more susceptible to damage (Primorac, Molnar et al. 2020). The persistent inflammatory response in KOA plays a role in the deterioration of cartilage and other joint components, heightening discomfort and functional limitations. Characteristic symptoms of KOA encompass knee pain, restricted knee movement, joint stiffness, muscle weakness, functional mobility impairments, and disturbances in postural balance (Liu, Luo et al. 2022).

2.2 Prevalence and Impact of KOA

Knee osteoarthritis (KOA), also referred to as degenerative joint disease, stands as a prominent cause of physical impairment and disability (Chan, Dittakan and Garcia-Constantino 2020, Peat and Thomas 2021). The global incidence and prevalence of KOA are on the rise, with an estimated 654 million individuals diagnosed with KOA in 2020 (Cui, Li et al. 2020). According to (Zamri, Harith et al. 2021), they conducted a study included knee and/or hip osteoarthritis (OA) patients, comprising 19.1% men and 80.9% women, with an average age of 61.81 years. Body weight distribution showed 1.5% underweight, 12.2% normal weight,

36.7% overweight, and 49.6% obese patients. The social functioning (Unver, Karatosun and Bakirhan) domain had the highest mean score (41.25), while the mental domain scored the lowest (21.15). Males had significantly greater weight and height but lower body fat compared to females, with higher energy, carbohydrate, and protein intake. Patients aged < 60 years exhibited significantly greater weight, height, and body fat compared to those aged ≥ 60 years.

Knee osteoarthritis (KOA) presents a multifaceted challenge, demanding comprehensive attention and intervention due to its significant impact on individuals, healthcare systems, and the global economy. Studies have indicated that the increasing prevalence of KOA places a substantial strain on the health system, particularly evident in primary care settings where most chronic conditions are managed (Ginnerup-Nielsen, Henriksen et al. 2019, Ji, Liu et al. 2023). Consequently, the considerable financial burden of osteoarthritis (OA) within primary care significantly exacerbates the global economic challenges encountered by health systems. An emphasized review points to increased rates of primary care consultations and hospital admissions among those with KOA, coupled with a heightened risk of all-cause mortality compared to age- and sex-matched controls (Swain, Coupland et al. 2023). This demonstrates KOA as a substantial cause of morbidity, highlighting the necessity of a comprehensive strategy for managing KOA, considering its effects on both primary care services and hospital admissions. Beyond the physical manifestations, KOA has the potential to lead to enduring disabilities, impacting both physical and mental health (Ahmad, Ajit Singh et al. 2018). Addressing psychological symptoms emerges as a crucial aspect of KOA treatment, with individuals experiencing pain often exhibiting long-term depression (Lu, Wang et al. 2022). The information above highlights the interconnectedness of physical and mental well-being in the context of KOA.

2.3 Current Management of KOA

The management of osteoarthritis (OA) involves a range of approaches, including non-regenerative methods like exercise, biomechanical interventions, electrotherapy, diathermy, and pharmacology. Exercise contributes to weight loss, improved function, and enhanced quality of life, while biomechanical interventions modify the onset and progression of OA (Jurado-Castro, Muñoz-López et al. 2022). Pharmacotherapy involves NSAIDs, with topical applications recommended. Intra-articular corticosteroid injections target inflammation, genicular nerve blocks and radiofrequency therapies are emerging interventions (Cao, Li et al. 2020). Regenerative options include laser therapy and platelet-rich plasma injections.

Prolotherapy, involving hypertonic dextrose injections, is explored for its potential in treating musculoskeletal pain. Each modality carries its own benefits and considerations in tailoring OA treatment strategies (Mintarjo, Poerwanto and Tedyanto 2023).

Recognizing effective treatment strategies for knee osteoarthritis (KOA) is imperative (Peat and Thomas 2021). Nonetheless, the absence of a definitive disease-modifying intervention for KOA prompts healthcare practitioners to integrate pharmacological measures and rehabilitation exercises with electro-modalities for enhanced outcomes (Collins, Hart and Mills 2019, Peat and Thomas 2021). The commonly employed electro-modalities encompass transcutaneous electrical stimulation (TENS), therapeutic ultrasound (US), and the relatively recent addition, high-intensity laser therapy (HILT) (Bannuru, Osani et al. 2019, Ahmad, MS and Yusof 2022). Intriguingly, recent research indicates that HILT may outperform TENS and US in effectively addressing pain and enhancing physical function among individuals with KOA (Samaan, Sedhom and Grace 2022, Wu and Zhu 2022).

2.4 Mechanism of HILT

Photobiomodulation, commonly known as laser therapy, represents a therapeutic approach employing therapeutic light doses to non-invasively address injured or dysfunctional tissue, thereby promoting pain relief and tissue healing through photo-biological mechanisms (White, Alvir-Lazo and Yumul 2019, Wickenheisser, Zywot et al. 2019, BTL-Corporate 2020). This technique relies on the absorption of laser energy (photons) by tissue and cells, initiating cellular mitochondrial oxidative reactions. This process leads to the production of adenosine triphosphate (ATP), crucial for optimal cell metabolism and healing, as well as the modulation of low-level reactive oxygen species and the release of nitric oxide—a potent vasodilator that aids in reducing pain and inflammation (Kushibiki and Ishihara 2017, White, Alvir-Lazo and Yumul 2019).

High-intensity laser therapy (HILT) emerges as an advanced iteration of laser therapy, distinguished by its capacity to deliver high energy output (exceeding 500 mW), penetrate deep tissues (up to 15 cm), and induce superficial hyperthermia through photothermic effects (BTL-Corporate 2020, Penberthy and Vorwaller 2021). HILT demonstrates the ability to augment oxidative processes and rapidly increase ATP synthesis (BTL-Corporate 2020). Notably, HILT, especially when utilizing wavelengths beyond 1064 nm, can directly target nerve endings, providing immediate pain relief (Penberthy and Vorwaller 2021). Due to its anti-inflammatory

properties, bio-stimulation effects, and photothermic advantages, HILT stands out as a promising therapeutic option for addressing knee osteoarthritis (KOA). This innovative approach holds potential for effectively managing KOA by leveraging its ability to enhance cellular processes, mitigate inflammation, and promptly alleviate pain through direct nerve targeting.

2.5 Clinical Evidence of HILT in KOA

While the therapeutic application of laser technology, specifically High-Intensity Laser Therapy (HILT), is thought to have a positive impact on knee osteoarthritis (KOA) by modulating pain and stimulating tissue (Stausholm, Naterstad et al. 2019, Penberthy and Vorwaller 2021, Ahmad, MS and Yusof 2022), its incorporation into current KOA treatment guidelines is notably absent due to the insufficient backing of clinical evidence (Brophy and Fillingham 2022, Overton, Nelson and Neogi 2022). Despite the existence of inconclusive evidence regarding the efficacy of HILT in managing KOA (Huang, Chen et al. 2015), previous trials are burdened with several limitations. Primarily, these trials predominantly explored the isolated effects of HILT without accounting for the influence of KOA rehabilitation exercise, which is recognized as the primary conservative treatment for KOA (Ahmad, MS and Yusof 2022). Secondly, past studies primarily focused on short-term effects, leaving a considerable gap in understanding the long-term effectiveness of HILT, especially considering KOA as a chronic disease (Penberthy and Vorwaller 2021, Ahmad, MS and Yusof 2022).

Moreover, the majority of existing literature has centered around the symptomatic management effects of HILT(Stausholm, Naterstad et al. 2019, Penberthy and Vorwaller 2021, Ahmad, MS and Yusof 2022), with uncertainties prevailing regarding its potential disease-modifying effects capable of reversing joint damage in KOA. Addressing these limitations becomes imperative in elucidating the comprehensive role of HILT in KOA management. Therefore, future research endeavors should emphasize a more integrated approach, considering the synergistic effects of HILT in conjunction with rehabilitation exercises to ascertain its full therapeutic potential for KOA. Additionally, there is a pressing need for more extended duration studies that can shed light on the prolonged efficacy of HILT in managing KOA, recognizing the chronic nature of the condition. Furthermore, investigations exploring the disease-modifying attributes of HILT, particularly its capacity to reverse joint damage in KOA, would significantly contribute to advancing our understanding of its therapeutic impact on this prevalent and challenging musculoskeletal disorder.

Currently, there is a deficiency in qualitative research on laser therapy. However, an investigation conducted by undergraduate Dentistry students delved into the application of diode laser in frenectomy surgeries. Semi-structured interviews were carried out one week post-surgeries and subsequently transcribed for content analysis. Thematic analysis uncovered three main themes: positive attitudes toward diode laser use, frustrations linked to its application, and considerations associated with professional training. The researchers provided evidence supporting the efficacy of laser therapy in reducing pain, expediting healing, and minimizing both transoperative and postoperative discomfort. (Soares, Carvalho et al. 2020)

Table 2.1. Clinical effects of HILT.

No	Study	Methodology	Findings
1.	(Penberthy and Vorwaller 2021) Utilization of the 1064 nm Wavelength in Photobiomodulation: A Systematic Review and Meta-Analysis	• Conducted a comprehensive search (2016-2021) in PubMed, Google Scholar, and Cochrane databases, specifically targeting clinical trials related to "photobiomodulation" with a focus on the 1064 nm wavelength and high-powered lasers (>0.5 watt).<br>• Employed Cohen's d to calculate effect size, assessing the relative efficacy of 1064 nm HPL-PBMT.	• Studies on 1064 nm HPL-PBMT demonstrated consistent pain reduction, improved function, and enhanced quality of life across diverse medical conditions such as knee arthropathies, spinal disorders, and more.<br>• Particularly effective in knee arthritis, 1064 nm HPL-PBMT showed statistically significant and sustained pain reduction, indicating potential as a non-invasive treatment option.
2.	(Stausholm, Naterstad et al. 2019)<br><br>Efficacy of low-level laser therapy on pain and disability in knee osteoarthritis: systematic review and meta-analysis of randomised placebo-controlled trials	• Study design: Systematic Review<br>• Utilized data from 22 randomized placebo-controlled trials (n=1063), synthesized with random effects meta-analyses and subgrouped by LLLT dose according to World Association for Laser Therapy recommendations.	• LLLT significantly reduced pain and disability in KOA, both at the end of therapy and during follow-ups 1–12 weeks later.<br>• Subgroup analysis highlighted significant pain reduction with recommended LLLT doses, peaking during follow-ups 2–4 weeks post-therapy.

			• No adverse events were reported, concluding that LLLT is effective in reducing pain and disability in KOA under specific dosage and wavelength conditions.
3.	(Huang, Chen et al. 2015)<br>Effectiveness of low-level laser therapy in patients with knee osteoarthritis: a systematic review and meta-analysis	• Systematically searched MEDLINE, EMBASE, ISI Web of Science, and Cochrane Library for relevant randomized controlled trials (RCTs) from January 2000 to November 2014, focusing on studies in English that compared LLLT (minimum eight sessions) with sham laser in KOA patients.	• Out of 612 studies, nine RCTs (518 patients) were included. The standardized mean difference (SMD) in visual analog scale (VAS) pain score right after therapy (within 2 weeks) did not significantly differ between LLLT and control. No significant differences were found in studies following World Association of Laser Therapy (WALT) recommendations or based on OA severity.
4.	(Overton, Nelson and Neogi 2022)<br>Osteoarthritis Treatment Guidelines from Six Professional Societies: Similarities and Differences	• Study design: Systematic Review<br>• A literature search was conducted in November 2021 to identify osteoarthritis (OA) guidelines published or updated within the past 5 years	• The OA treatment guidelines from 6 professional societies show substantial agreement, indicating a consistent approach to osteoarthritis management across diverse healthcare organizations.

5	(Samaan, Sedhom and Grace 2022)<br>A randomized comparative study between high-intensity laser vs low-intensity pulsed ultrasound both combined with exercises for the treatment of knee osteoarthritis	• Study design: RCT<br>• No. of participants: 60<br>• Participants with grades II and III KOA, randomly assigned to three groups: HILT+ET, LIPUS+ET, and a control group receiving only ET. Treatment was administered five times per week for two consecutive weeks.	• HILT+E demonstrated superior improvement in pain, knee range of motion (ROM), proprioceptive accuracy, and functional disability compared to LIPUS+E and the control group. Statistical analyses revealed significant time-by-group effects across all outcomes, with HILT+E outperforming both LIPUS+E and the control group. The findings suggest that the combined approach of HILT with exercises yields better results than LIPUS combined with exercises, and both are more effective than exercises alone in managing knee osteoarthritis.

RCT: Randomized Controlled trial; LPT: High Pain Threshold; LPT: Low Pain Threshold; LLLT :Low level Laser Therapy; WALT: World Association of Laser Therapy; ROM: Range of motion; VAS: Visual Analog Scale.

2.6 Summary of Literature Findings

The literature review aims to comprehensively explore the pathophysiology of knee osteoarthritis (KOA), its prevalence, and the subsequent impact on both physical and psychological health. It encompasses an in-depth examination of the mechanism, prevalence, and consequences of KOA, leading to a discussion on current management strategies and a focus on laser therapy as an alternative approach. The review delves into the principles of laser therapy, evaluating clinical evidence regarding its effects on pain, physical function, and psychological health. Notably, it concludes by summarizing key findings and emphasizing existing knowledge gaps, providing a foundation for future studies. The subsequent sections detail the pathophysiology of KOA, its prevalence and impact, current management approaches, the mechanism of High-Intensity Laser Therapy (HILT), and clinical evidence of HILT in KOA.

The review recognizes the interconnectedness of physical and mental well-being in the context of KOA, emphasizing the multifaceted challenge it poses and the necessity for a comprehensive management strategy. It further underscores the potential of HILT as a promising therapeutic option for addressing KOA, calling for more integrated and extended-duration studies to elucidate its full therapeutic potential. Additionally, it addresses the lack of qualitative research on laser therapy, citing a study by undergraduate Dentistry students exploring the application of diode laser in frenectomy surgeries and providing evidence supporting its efficacy in reducing pain and facilitating healing.

3.0 Methodology

The proposed study will consist of 3 phases:

- Phase 1: Quantitative design- Randomized Controlled Trial (Short and long-term outcomes)
- Phase 2: Qualitative study- Structured one-on=one interviews (Participants' experiences and perspectives)

3.1 Phase 1: Quantitative Design-Randomized Controlled Trial (Short-term Outcomes)

3.1.1 Study Design

This prospective investigation will adopt a parallel-group, randomized, double-blinded (for both subjects and outcomes assessor), and placebo-controlled trial design. The study protocol adheres to the guidelines outlined in the Consolidated Standards of Reporting Trials (CONSORT). Participants will be randomly assigned to either the High-Intensity Laser Therapy with exercise (HILT+E; n = 20) group or the placebo laser with exercise (PL+E; n = 20) group.

3.1.2 Study Settings

The research will take place at Hospital Canselor Tuanku Muhriz UKM, Malaysia, where adults in the early stages of Knee Osteoarthritis (KOA) will undergo screening and recruitment from the Physiotherapy Department, ensuring they meet the specified inclusion and exclusion criteria.

3.1.3 Target Population

The intended participants for this research will be adults aged 18 years and older who are in the early stages of Knee Osteoarthritis (KOA). The inclusive and exclusive criteria will encompass:

3.1.3.1 Inclusion Criteria

i. Adults aged 18 years and above of both sexes.
ii. Individuals diagnosed with unilateral or bilateral KOA by an orthopaedic doctor.
iii. Those with a Kellgren-Lawrence classification of grade ≤ 2 (mild) based on a knee radiograph conducted within the last six months

iv. Individuals capable of engaging in the intervention and assessment program without restrictions. In cases where both knee joints are affected, the knee with more severe symptoms, based on the pain score, will be included. If symptoms are identical in both knees, the dominant knee will be assessed.

v. Individuals who can understand and express themselves in English and Bahasa Malaysia.

3.1.3.2 Exclusion criteria

i. Presence of other pathological conditions like rheumatic disease, prior hip or knee joint replacement, congenital dysplasia, osteochondritis dissecans, intra-articular fractures, septic arthritis, ligament or meniscus injury, and any other condition impeding the healing process, including diabetes mellitus and peripheral vascular disease

ii. comorbidities hindering physical evaluation

iii. ongoing participation in another KOA-related interventional study

iv. individuals taking prescription glucosamine sulfate, which might potentially affect the study's outcomes.

v. Participants who had undergone intra-articular knee injections (e.g., corticosteroid, hyaluronic acid, or blood-derived products) for KOA management in the last six months

vi. Individuals who unable to understand and express themselves in English and Bahasa Malaysia.

3.1.4 Sample Size Calculation

The determination of the sample size for this study will be conducted using G*Power software version 3.1.9.7, relying on a minimal clinically important difference (MCID) of 11 points for the primary outcome, Knee Injury and Osteoarthritis Outcome Score (KOOS), as indicated by (Eckhard, Munir et al. 2021). We will consider a pre-specified power of 90%, an effect size of 0.3, an alpha level of 5%, and a potential dropout rate of 20%, following the approach outlined by (Serdar, Cihan et al. 2021). Consequently, the necessary total sample size will be 40, with an allocation of 20 participants to each group.

3.1.5 Sampling and randomization

The screening, recruitment, and randomization (group allocation) of participants will be carried out by a researcher uninvolved in the intervention or outcomes assessment. After

recruiting 40 participants, they will be randomly assigned in a 1:1 ratio to one of two intervention groups using a computer-generated table and a simple block randomization method: (i) Group HILT with exercise (HILT+E; n = 20) and (ii) Group placebo laser with exercise (PL+E; n = 20). This randomization process will occur subsequent to participants receiving both verbal and written information about the study protocol and signing the written informed consent form.

3.1.6 Group allocation and intervention

In this research, participants will be randomly assigned into one of two groups:

- Group 1 (HILT+E): Participants from this group will undergo individually tailored standard physiotherapy alongside high-intensity laser therapy for twelve weekly sessions. The treatment session will be administered by a qualified physiotherapist who has undergone training in prescribing laser therapy.
- Group 2 (PL+E): Participants will receive individually tailored standard physiotherapy with a placebo/sham intervention once a week for twelve sessions.

Participants in both Group HILT+E and Group PL+E will receive HILT (BTL-6000: 5 W, 1064 nm, 19 to 150 J/cm2, and 3190 J per session) and sham laser (without emission of energy) as an adjunctive treatment, respectively, in addition to knee rehabilitation exercise (standard physiotherapy care). The specifics of laser interventions based on group allocation are outlined in the attached Appendix, with parameters and procedures derived from manufacturer guidelines(BTL-Corporate, 2020) and previous studies(Kheshie et al., 2014; Ahmad Nazari et al., 2019). To ensure participant blinding regarding their laser intervention, the laser procedures will be identical between groups, including visual light and acoustic indicators (de Paiva et al., 2019). Despite laser therapy being generally safe with few reported adverse effects (Rayegani et al., 2017), this study will implement specific safety measures and precautions. Participants will be required to wear special protective eyewear during laser application, and they will be periodically checked for any discomfort or overheating. Additionally, participants will be asked to report any adverse effects of the laser intervention during the weekly follow-up session.

Qualified physiotherapists from the Hospital Canselor Tuanku Muhriz UKM, unaware of the participants' laser grouping, will administer the personalized physiotherapy treatment,

adhering to recommended KOA treatment guidelines. The knee rehabilitation exercises utilized will be adapted from pertinent previous studies(Gay et al., 2016; Suzuki et al., 2019) and the recommended KOA treatment guidelines (Bannuru et al., 2019; Collins et al., 2019). Each session is anticipated to last one hour, encompassing various knee exercises. The exercise prescription and progression will be modified accordingly based on the individual patient's KOA. The examples of exercise components are:

- Range of motion
  - Prone knee bend (2 set, 10 repetitions each)
  - Supine alternate knee bend (2 set, 10 repetitions each)
- Stretching
  - Standing quadriceps stretch (1 set, 3-5 repetitions, 15 second hold each)
  - Calf stretch in long sitting (1 set, 3-5 repetitions, 15 second hold each)
  - Supine hamstring stretch (1 set, 3-5 repetitions, 15 second hold each)
- Strengthening
  - Sitting knee extension (Week 1-2: 1 set, 5 repetitions, 5-second hold; Week 3-5: 1 set, 7-10 repetitions, 5-10-second hold; Week 6-8: 2 sets, 10 repetitions, 10-second hold)
  - Supine straight leg raise (Week 1-2: 1 set, 5 repetitions, 5-second hold; Week 3-5: 1 set, 7-10 repetitions, 5-10-second hold; Week 6-8: 2 sets, 10 repetitions, 10-second hold)
  - Static quadriceps (Week 1-2: 1 set, 5 repetitions, 5-second hold; Week 3-5: 1 set, 7-10 repetitions, 5-10-second hold; Week 6-8: 2 sets, 10 repetitions, 10-second hold)
  - Side-lying straight leg raise (Week 1-2: 1 set, 5 repetitions, 5-second hold; Week 3-5: 1 set, 7-10 repetitions, 5-10-second hold; Week 6-8: 2 sets, 10 repetitions, 10-second hold)
- Functional Training
  - Sit-to-stand (10 repetitions)
  - Standing mini squat (10 repetitions)
  - Walking exercise (Approximately 2 minutes for each item)

3.1.7 Outcome Measures

In this phase of the study, outcome measures will be evaluated at two distinct intervals:

1. Baseline (pre-intervention): These assessments are conducted before the intervention to establish the initial state of participants' KOA condition.
2. Immediately post-completion of the intervention (short term, week-8): These measurements are taken immediately after the last intervention, occurring at week 8.
3. Follow-up (long-term, week-12): The assessments are conducted after the intervention occurring three months after the final treatment session at week 24.

All outcomes will be assessed by a blinded assessor not involved in carrying out the intervention. Additionally, participants will be screened for potential adverse events, such as bruising, fatigue, weakness, and swelling, during each follow-up visit throughout the treatment. The outcomes to be measured in the study include:

1. Knee Injury and Osteoarthritis Outcome Score (KOOS): This validated questionnaire assesses the outcomes and subjective experiences of individuals with knee injuries or osteoarthritis, encompassing domains like pain, symptoms, activities of daily living, sports and recreation, and quality of life. The KOOS demonstrates high reliability and validity in different languages, with excellent test-retest reliability (intraclass correlation coefficients ranging from 0.91 to 0.99)(Phatama et al., 2021). Please refer to the Appendix 1.
2. Active knee flexion Range of Motion (ROM): Knee flexion will be measured using a goniometer, with high reliability (intraclass correlation coefficient (ICC) of 0.997 for knee flexion)(Remigio et al., 2017). The goniometer is reliable for evaluating knee flexion in various positions, including supine, prone, and sitting positions, with highly correlated results ($r > 0.80$) (Unver et al., 2009).
3. Timed Up and Go (TUG) test: This test assesses functional mobility by measuring the time required to stand from a chair, walk 3 meters, and sit again. The TUG test is chosen for its high reliability and validity in Grade 1-3 KOA, with a minimal detectable change of 1.10 seconds (Chen et al., 2022). OARSI recommends the TUG test for KOA patients, with strong intra-rater and inter-rater reliability values (0.97 and 0.96, respectively) (Alghadir et al., 2015).
4. Numeric Pain Rating Scale (NPRS): This is a tool commonly used to assess pain intensity, utilizing an 11-point scale ranging from 0-10. In this scale, 0 signifies “no pain” while 10

represent the “worst imaginable pain”. The individual are asked to select a single number from this scale that most accurately reflects their current level of pain. A study reported excellent reliability for NPRS with ICC of 0.95.(Alghadir et al., 2018)

5. Knee radiographic imaging, involving X-ray examinations, will be employed to quantitatively assess changes in knee joint structure damage. The examination will only be conducted during baseline and follow-up interval. A qualified radiologist will conduct the knee X-ray examinations to ensure standardized assessments and minimize assessor bias (Lopes et al., 2023) . The evaluation will focus on key parameters, including joint space width, which measures the distance between the femur and tibia to assess joint space narrowing; joint alignment, involving the assessment of angles like the mechanical axis of the leg to identify varus or valgus deformities; and cartilage thickness, measuring articular cartilage thickness in specific knee joint regions to monitor cartilage loss.

   The utilization of X-rays as an outcome measure aims to evaluate the severity and progression of KOA by examining changes in joint space, the presence of osteophytes, and the degree of bone or joint destruction(Yang et al., 2022). X-rays are a widely used diagnostic tool for KOA in clinical practice, offering an objective measure of disease progression over time (El-Ghany et al., 2023). The choice of X-rays is practical due to their relatively low cost and widespread availability in clinical and research settings. Extensive research has demonstrated the reliability and validity of using X-rays to assess KOA, showing high inter-observer reliability and moderate-to-high validity compared to other imaging modalities such as MRI or ultrasound (Paraskevas et al., 2023).

3.1.8 Data Analysis

The data analysis for this study will be conducted using SPSS version 25.0 (SPSS Inc., Chicago, IL, USA) and will adhere to the intention-to-treat principle. All participants, irrespective of their adherence to the intervention, will be included in the analysis. Sociodemographic characteristics of participants at baseline will be described using descriptive statistics and cross-tabulations. The primary statistical analysis for assessing outcomes will involve repeated measures ANOVA, contingent upon meeting assumptions of normality and sphericity. Normality will be evaluated using the Shapiro-Wilk test, with higher values indicating more normal data, while sphericity will be assessed using Mauchly’s test, where lower values closer to 0 suggest more homogenous variance between levels.

The data will undergo two-way repeated measure ANOVA to scrutinize the effects of time, group, and the interaction between the two investigated groups (HILT+E and PL+E). Cohen's d will be applied to ascertain the effect size of each variable, with values of 0.2, 0.5, and 0.8 representing small, medium, and large effects, respectively (Serdar, Cihan et al. 2021). Moreover, the Minimal Clinically Important Difference (MCID) will be considered for the KOOS, NPRS, active knee flexion range, and TUG test to evaluate the clinical relevance of observed changes(Eckhard et al., 2021; Maldaner et al., 2022; Salaffi et al., 2004). All statistical analyses will maintain an alpha level of 0.05 for significance tests (Serdar et al., 2021).

3.2 Phase 2: Qualitative Study-Structured One-On-One Interviews (Participants' Psychological Well-being, Experiences, and Perspectives)

During the third phase of our research, we aim to qualitatively investigate the perspectives and experiences of adults with KOA who have undergone a 12-week intervention involving HILT with exercise, in comparison to those who received placebo laser therapy with exercise.

3.2.1 Procedures

To accomplish this goal, the study will conduct individual interviews either in person or online, using a structured research questionnaire. We will arrange suitable physical or online meeting locations, ensuring participants' comfort with their chosen method. The interviews will be conducted on a one-on-one basis with selected participants, and each interview will be audio or video-recorded with participants' consent. A trained interviewer, knowledgeable about the study's objectives but maintaining neutrality to prevent bias, will administer the interviews. Questions will be posed in a conversational manner, allowing participants to elaborate on their experiences and perspectives. Comprehensive notes, capturing both verbal responses and non-verbal cues, will be taken during the interviews.

3.2.2 Sampling method

Total of 10 participants from each group will be randomly selected using a computer-generated list to mitigate any potential selection bias. The sampling process will be performed by the principal investigator, who will not be involved in the intervention and outcome assessment. Sampling will continue until thematic saturation is reached, ensuring comprehensive coverage of the research topic.

3.2.3 Outcomes Measures

The outcomes of interest include participants' psychological well-being, experiences, and perspectives throughout the 12-week intervention involving HILT with exercise or placebo laser therapy with exercise. These outcomes will be explored through structured one-on-one interview, which allowing participants to share their subjective experiences and perceptions. The interview script is generated based on WHO-5 Well-Being Index and SF-36.

1. WHO-5 Well-Being Index: Developed by the World Health Organization, this index assesses overall psychological well-being by querying mood, vitality, and general well-being. The WHO-5 demonstrates strong internal consistency (Cronbach's alpha coefficient of 0.858) and convergent validity where the significant correlations with PHQ-9, HADS-anxiety, and HADS-depression (Maroufizadeh et al., 2019). Please refer to the Appendix 2.
2. Short Form 36 Health Survey (SF-36): Widely used to assess health-related quality of life, SF-36 provides valuable insights into psychological well-being. Despite being primarily designed for health-related quality of life, SF-36's total scores exhibit high responsiveness (>0.9 effect sizes) (Clement et al., 2022). Thresholds of ≤52 in the MH subscale and ≤56 in the SF-36 effectively detect anxiety and depression, with additional reliable thresholds (≤35 and ≤40 in MCS scores) for identifying these conditions (Matcham et al., 2016). Please refer to the Appendix 3.

Interview Script

1) How has the intervention, specifically the physiotherapy management, impacted your experience with knee osteoarthritis?
2) Feel free to discuss any challenges or successes you've encountered during the intervention, and whether your expectations were met.
3) Can you describe any noticeable improvements in your knee pain or related issues since the start of the intervention?
4) How do you perceive the impact of the intervention on your overall quality of life?
5) How would you describe your emotional well-being in relation to your knee osteoarthritis after undergoing the intervention?

3.2.4 Data Analysis

The qualitative data gathered from individual interviews will undergo thematic analysis using ATLAS.ti software programs. Thematic analysis is employed to achieve a profound understanding of participants' perspectives and experiences, providing valuable insights to the study. This analysis involves multiple stages, starting with the transcription of interview recordings into text. The transcribed data is then imported into the ATLAS.ti software for detailed examination. The process includes data familiarization, where interview transcripts are reviewed to become well-acquainted with their content. Codes are developed to categorize various segments of the data, and patterns and themes are identified from the coded data. To ensure accuracy and consistency, these themes are refined and validated through comparison across different interviews, ensuring they accurately represent participants' viewpoints.

3.3.3 Ethic consideration and participation confidentially

Research must adhere to ethical guidelines to safeguard the rights and well-being of both researchers and participants. Participants should feel free to voluntarily engage in the study, with the freedom to withdraw at any time if they feel insecure or unsafe during the course of the research. Prior to commencement, participants are provided with and required to read an informed consent form, which outlines potential risks and benefits associated with participation. The research proceeds only upon agreement from the participants.

Data collected is solely utilized for educational purposes, with strict measures in place to ensure the security and confidentiality of participants' identities, facilitated by secure servers. Data will be promptly deleted when it is no longer necessary for the study. Furthermore, researchers refrain from discussing research findings in public forums and remove any identifying information from databases containing sensitive data.

3.3.4 Budget

The study has been approved by Geran Galakan Penyelidik Muda (GGPM) with code number GGPM-2023-077 on 5 October 2023. The total funding for this project is RM40,000.00.

Table 3.0: Budget Details

Budget details	Description	Total (RM)
Wages and allowances	Appoint a Graduate Research Assistant (GRA)	10,200.00

	RM1700/month x 6 months	
Travel and transportation (40% of total projek amount expect the project Dana Cabaran Perdana 25% of the total project amount.	Domestic travel.<br>(1) Researchers (travel, transportation and parking fees)=RM 800<br>(2) Participants (travel and transportation): RM10/ session x 8 sessions x 40 participants = RM 3200	4000.00
Rental	Medical equipment rental<br>Rental of BTL-6000 High-intensity laser 12W machine from BTL Industries Malaysia Sdn. Bhd.: RM1000/month x 6 months	6000.00
Research supplies and materials	Consumables: Wipes and disinfectants	1400.00
	ICT inventory-less than RM3000<br>Printer and electronic storage (External hard disk)	
Minor repair and alterations	-	-
Specialized services	Professional service fee<br>(1) Enumerator (interpretation of knee radiographic X-ray): RM 50/hours x 10 hours=RM 500<br>(2) Physiotherapist (upon completion of the study): RM 500 x 1 physiotherapist= RM500	1000.00
	Sample analysis fees.<br>Outcome measure- knee radiographic imaging (X-ray): RM100/investigation x 2 (baseline week-0 and follow-up at week-24) x 40 participants	8000.00
	Fee payment:	9400.00

	(1) Conference and journal article publication fee payment (Open access – WOS Q1/Q2)=RM 9000<br>(2) Ethic application and clinical trial coverage (2 years) =RM 400	
	Total:	18,400.00
Accessories and equipment (40% of the total amount of the project)	-	-
**Total amount of the project**		40,000.00

3.3.5 Flow Chart of Trial

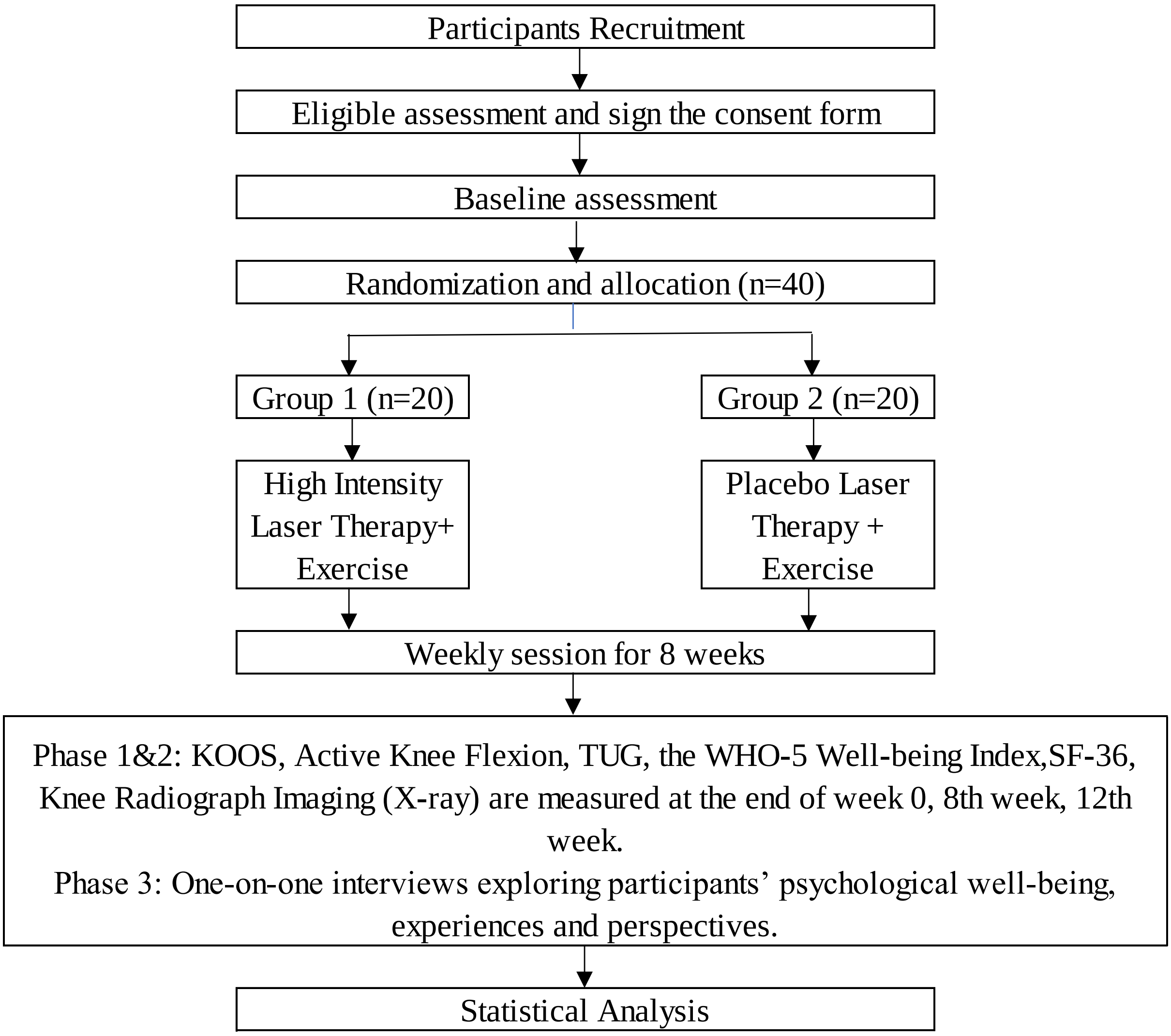

Gantt Chart

Activity	Year 1 2024												Year 2 2025											
	1	2	3	4	5	6	7	8	9	10	11	12	1	2	3	4	5	6	7	8	9	10	11	12
Ethics application and trial protocol registration																								
Research materials and equipment procurement: Acugraph system																								
Screening, recruitment, randomization & intervention																								
Data collection (short-term effects)-Phase 1																								
Data collection (follow-up assessment) - Phase 2																								
Data collection - Phase 3																								

Data analysis																								
Reporting the findings and journal article publication																								

4.0 Reference list

1. Ahmad, M. A., D. K. Ajit Singh, C. W. Qing, N. N. A. Ab. Rahman, F. A. Mohd Padzil and E. N. Muhammad Hendri (2018). "Knee Osteoarthritis and Its Related Issues: Patients' Perspective." Jurnal Sains Kesihatan Malaysia (Malaysian Journal of Health Sciences); Vol 16 (2018): Special Issue (Faculty of Health Science Silver Jubilee 2017).
2. Ahmad, M. A., M. Moganan, M. S. A Hamid, N. Sulaiman, U. Moorthy, N. Hasnan and A. Yusof (2023). "Comparison between Low-Level and High-Intensity Laser Therapy as an Adjunctive Treatment for Knee Osteoarthritis: A Randomized, Double-Blind Clinical Trial." Life **13**(7): 1519.
3. Ahmad, M. A., A. H. MS and A. Yusof (2022). "Effects of low-level and high-intensity laser therapy as adjunctive to rehabilitation exercise on pain, stiffness and function in knee osteoarthritis: a systematic review and meta-analysis." Physiotherapy **114**: 85-95.
4. Alghadir, A., S. Anwer and J.-M. Brismée (2015). "The reliability and minimal detectable change of Timed Up and Go test in individuals with grade 1 – 3 knee osteoarthritis." BMC musculoskeletal disorders **16**: 174.
5. Bannuru, R. R., M. C. Osani, E. E. Vaysbrot, N. K. Arden, K. Bennell, S. M. A. Bierma-Zeinstra, V. B. Kraus, L. S. Lohmander, J. H. Abbott, M. Bhandari, F. J. Blanco, R. Espinosa, I. K. Haugen, J. Lin, L. A. Mandl, E. Moilanen, N. Nakamura, L. Snyder-Mackler, T. Trojian, M. Underwood and T. E. McAlindon (2019). "OARSI guidelines for the non-surgical management of knee, hip, and polyarticular osteoarthritis." Osteoarthritis Cartilage **27**(11): 1578-1589.
6. Brophy, R. H. and Y. A. Fillingham (2022). "AAOS Clinical Practice Guideline Summary: Management of Osteoarthritis of the Knee (Nonarthroplasty), Third Edition." J Am Acad Orthop Surg **30**(9): e721-e729.
7. BTL-Corporate. (2020). "High Intensity Laser: painless and non-surgical management." Retrieved 15 February 2020, from www.high-intensity-laser.com.
8. Cao, P., Y. Li, Y. Tang, C. Ding and D. J. Hunter (2020). "Pharmacotherapy for knee osteoarthritis: current and emerging therapies." Expert Opin Pharmacother **21**(7): 797-809.
9. Chan, S., K. Dittakan and M. Garcia-Constantino (2020). "Image Texture Analysis for Medical Image Mining: A Comparative Study Direct to Osteoarthritis Classification using Knee X-ray Image." International Journal on Advanced Science Engineering and Information Technology **10**.

10. Chen, H., C. Wang, J. Wu, M. Wang, S. Wang, X. Wang, J. Wang, H. Yu, Y. Hu and S. Shang (2022). "Measurement properties of performance-based measures to assess physical function in knee osteoarthritis: A systematic review." Clinical Rehabilitation **36**(11): 1489-1511.
11. Clement, N. D., D. Weir and D. Deehan (2022). "Meaningful values in the Short Form Health Survey-36 after total knee arthroplasty - an alternative to the EuroQol five-dimension index as a measure for health-related quality of life : minimal clinically important difference, minimal important change, patient-acceptable symptom state thresholds, and responsiveness." Bone Joint Res **11**(7): 477-483.
12. Collins, N. J., H. F. Hart and K. A. G. Mills (2019). "Osteoarthritis year in review 2018: rehabilitation and outcomes." Osteoarthritis Cartilage **27**(3): 378-391.
13. Cui, A., H. Li, D. Wang, J. Zhong, Y. Chen and H. Lu (2020). "Global, regional prevalence, incidence and risk factors of knee osteoarthritis in population-based studies." EClinicalMedicine **29-30**: 100587.
14. Du, X., Z.-y. Liu, X.-x. Tao, Y.-l. Mei, D.-q. Zhou, K. Cheng, S.-l. Gao, H.-y. Shi, C. Song and X.-m. Zhang (2023). "Research Progress on the Pathogenesis of Knee Osteoarthritis." Orthopaedic Surgery **15**(9): 2213-2224.
15. Eckhard, L., S. Munir, D. Wood, S. Talbot, R. Brighton, W. L. Walter and J. Baré (2021). "Minimal important change and minimum clinically important difference values of the KOOS-12 after total knee arthroplasty." Knee **29**: 541-546.
16. El-Ghany, S. A., M. Elmogy and A. A. A. El-Aziz (2023). "A fully automatic fine tuned deep learning model for knee osteoarthritis detection and progression analysis." Egyptian Informatics Journal **24**(2): 229-240.
17. Gay, C., A. Chabaud, E. Guilley and E. Coudeyre (2016). "Educating patients about the benefits of physical activity and exercise for their hip and knee osteoarthritis. Systematic literature review." Ann Phys Rehabil Med **59**(3): 174-183.
18. Geng, R., J. Li, C. Yu, C. Zhang, F. Chen, J. Chen, H. Ni, J. Wang, K. Kang, Z. Wei, Y. Xu and T. Jin (2023). "Knee osteoarthritis: Current status and research progress in treatment (Review)." Exp Ther Med **26**(4): 481.
19. Ginnerup-Nielsen, E., M. Henriksen, R. Christensen, B. L. Heitmann, R. D. Altman, L. March, A. D. Woolf, H. Karlsen and H. Bliddal (2019). University of Southern Denmark Prevalence of self-reported knee symptoms and management strategies among elderly individuals from Frederiksberg municipality Protocol for a prospective and pragmatic Danish cohort study.

20. Huang, Z., J. Chen, J. Ma, B. Shen, F. Pei and V. B. Kraus (2015). "Effectiveness of low-level laser therapy in patients with knee osteoarthritis: a systematic review and meta-analysis." Osteoarthritis Cartilage **23**(9): 1437-1444.
21. Ji, S., L. Liu, J. Li, G. Zhao, Y. Cai, Y. Dong, J. Wang and S. Wu (2023). "Prevalence and factors associated with knee osteoarthritis among middle-aged and elderly individuals in rural Tianjin: a population-based cross-sectional study." J Orthop Surg Res **18**(1): 266.
22. Jurado-Castro, J. M., M. Muñoz-López, A. S. Ledesma and A. Ranchal-Sanchez (2022). "Effectiveness of Exercise in Patients with Overweight or Obesity Suffering from Knee Osteoarthritis: A Systematic Review and Meta-Analysis." Int J Environ Res Public Health **19**(17).
23. Kheshie, A. R., M. S. Alayat and M. M. Ali (2014). "High-intensity versus low-level laser therapy in the treatment of patients with knee osteoarthritis: a randomized controlled trial." Lasers Med Sci **29**(4): 1371-1376.
24. Kushibiki, T. and M. Ishihara (2017). Biological function of low reactive level laser therapy (LLLT). Photomedicine - Advances in Clinical Practice. Y. Tanaka. London, IntechOpen**:** 198-213.
25. Liu, L., P. Luo, M. Yang, J. Wang, W. Hou and P. Xu (2022). "The role of oxidative stress in the development of knee osteoarthritis: A comprehensive research review." Front Mol Biosci **9**: 1001212.
26. Lopes, C., A. Vilaca, C. Rocha and J. Mendes (2023). "Knee positioning systems for X-ray environment: a literature review." Phys Eng Sci Med **46**(1): 45-55.
27. Lu, H., L. Wang, W. Zhou, S. Jin, H. Chen, Y. Su, N. Li and S. Shang (2022). "Bidirectional association between knee osteoarthritis and depressive symptoms: evidence from a nationwide population-based cohort." BMC Musculoskeletal Disorders **23**(1): 213.
28. Lv, Z., Y. X. Yang, J. Li, Y. Fei, H. Guo, Z. Sun, J. Lu, X. Xu, Q. Jiang, S. Ikegawa and D. Shi (2021). "Molecular Classification of Knee Osteoarthritis." Front Cell Dev Biol **9**: 725568.
29. Maldaner, N., M. Sosnova, M. Ziga, A. M. Zeitlberger, O. Bozinov, O. P. Gautschi, A. Weyerbrock, L. Regli and M. N. Stienen (2022). "External Validation of the Minimum Clinically Important Difference in the Timed-up-and-go Test After Surgery for Lumbar Degenerative Disc Disease." Spine (Phila Pa 1976) **47**(4): 337-342.

30. Maroufizadeh, S., R. Omani-Samani, A. Almasi-Hashiani, P. Amini and M. Sepidarkish (2019). "The reliability and validity of the Patient Health Questionnaire-9 (PHQ-9) and PHQ-2 in patients with infertility." Reprod Health **16**(1): 137.
31. Matcham, F., S. Norton, S. Steer and M. Hotopf (2016). "Usefulness of the SF-36 Health Survey in screening for depressive and anxiety disorders in rheumatoid arthritis." BMC Musculoskelet Disord **17**: 224.
32. Mintarjo, J. A., E. Poerwanto and E. H. Tedyanto (2023). "Current Non-surgical Management of Knee Osteoarthritis." Cureus **15**(6): e40966.
33. Nazari, A. and A. Moezy (2019). "Efficacy of high-intensity laser therapy in comparison with conventional physiotherapy and exercise therapy on pain and function of patients with knee osteoarthritis: a randomized controlled trial with 12-week follow up." **34**(3): 505-516.
34. Overton, C., A. E. Nelson and T. Neogi (2022). "Osteoarthritis Treatment Guidelines from Six Professional Societies: Similarities and Differences." Rheum Dis Clin North Am **48**(3): 637-657.
35. Paiva, P. R. V., H. L. Casalechi, S. S. Tomazoni, C. d. S. M. Machado, A. A. Vanin, B. M. Baroni, P. d. T. C. de Carvalho and E. C. P. Leal-Junior (2019). "Effects of photobiomodulation therapy combined to static magnetic field in strength training and detraining in humans: protocol for a randomised placebo-controlled trial." BMJ Open **9**(10): e030194.
36. Paraskevas, T., P. M. Dimopoulos, A. Kantanis, A. S. Garatzioti, I. Karalis, C. Michailides, C. Chourpiliadi, E. Matthaiakaki, C. Kalogeropoulou and D. Velissaris (2023). "Evaluation of Reliability and Validity of the RALE and BRIXIA Chest-X Ray Scores in Patients Hospitalized with COVID-19 Pneumonia." Rom J Intern Med **61**(3): 141-146.
37. Peat, G. and M. J. Thomas (2021). "Osteoarthritis year in review 2020: epidemiology & therapy." Osteoarthritis and Cartilage **29**(2): 180-189.
38. Penberthy, W. T. and C. E. Vorwaller (2021). "Utilization of the 1064 nm Wavelength in Photobiomodulation: A Systematic Review and Meta-Analysis." J Lasers Med Sci **12**: e86.
39. Peters, P. G., M. A. Herbenick, P. A. Anloague, R. J. Markert and L. J. Rubino, 3rd (2011). "Knee range of motion: reliability and agreement of 3 measurement methods." Am J Orthop (Belle Mead NJ) **40**(12): E249-252.

40. Phatama, K. Y., A. Aziz, M. H. Bimadi, I. Oktafandi, F. Cendikiawan and E. Mustamsir (2021). "Knee Injury and Osteoarthritis Outcome Score: Validity and Reliability of an Indonesian Version." Ochsner J **21**(1): 63-67.
41. Primorac, D., V. Molnar, E. Rod, Ž. Jeleč, F. Čukelj, V. Matišić, T. Vrdoljak, D. Hudetz, H. Hajsok and I. Borić (2020). "Knee Osteoarthritis: A Review of Pathogenesis and State-Of-The-Art Non-Operative Therapeutic Considerations." Genes (Basel) **11**(8).
42. Rayegani, S. M., S. A. Raeissadat, S. Heidari and M. Moradi-Joo (2017). "Safety and Effectiveness of Low-Level Laser Therapy in Patients With Knee Osteoarthritis: A Systematic Review and Meta-analysis." J Lasers Med Sci **8**(Suppl 1): S12-s19.
43. Remigio, W., N. Tsai, L. Layos and M. Chavez (2017). "Inter-rater and intra-rater reliability of the fluid goniometer for measuring active knee flexion in painful knees; correlations do not mean agreement." J Phys Ther Sci **29**(6): 984-988.
44. Salaffi, F., A. Stancati, C. A. Silvestri, A. Ciapetti and W. Grassi (2004). "Minimal clinically important changes in chronic musculoskeletal pain intensity measured on a numerical rating scale." Eur J Pain **8**(4): 283-291.
45. Samaan, S., M. G. Sedhom and M. O. Grace (2022). "A randomized comparative study between high-intensity laser vs low-intensity pulsed ultrasound both combined with exercises for the treatment of knee osteoarthritis." Int J Rheum Dis **25**(8): 877-886.
46. Serdar, C. C., M. Cihan, D. Yücel and M. A. Serdar (2021). "Sample size, power and effect size revisited: simplified and practical approaches in pre-clinical, clinical and laboratory studies." Biochem Med (Zagreb) **31**(1): 010502.
47. Siriratna, P., C. Ratanasutiranont, T. Manissorn, N. Santiniyom and W. Chira-Adisai (2022). "Short-Term Efficacy of High-Intensity Laser Therapy in Alleviating Pain in Patients with Knee Osteoarthritis: A Single-Blind Randomised Controlled Trial." Pain Res Manag **2022**: 1319165.
48. Soares Fonseca, L., J. Pereira Silva, M. Bastos Souza, M. Gabrich Moraes Campos, R. de Oliveira Mascarenhas, H. de Jesus Silva, L. Souza Máximo Pereira, M. Xavier Oliveira and V. Cunha Oliveira (2023). "Effectiveness of pharmacological and non-pharmacological therapy on pain intensity and disability in older people with chronic nonspecific low back pain: a systematic review with meta-analysis." European Spine Journal **32**(9): 3245-3271.
49. Soares, K. G., T. Y. A. d. Carvalho, A. M. C. Santos, L. B. Silveira, L. C. M. Costa, M. L. d. M. F. Fernandes and A. M. Fernandes (2020). "Perceptions of the Use of the Diode

Laser in Dental Surgery: A Qualitative Study." Pesquisa Brasileira em Odontopediatria e Clínica Integrada **20**.

50. Stausholm, M. B., I. F. Naterstad, J. Joensen, R. Á. B. Lopes-Martins, H. Sebo, H. Lund, K. V. Fersum and J. M. Bjordal (2019). "Efficacy of low-level laser therapy on pain and disability in knee osteoarthritis: Systematic review and meta-analysis of randomised placebo-controlled trials." BMJ Open **9**(10): e031142.
51. Suzuki, Y., H. Iijima, Y. Tashiro, Y. Kajiwara, H. Zeidan, K. Shimoura, Y. Nishida, T. Bito, K. Nakai, M. Tatsumi, S. Yoshimi, T. Tsuboyama and T. Aoyama (2019). "Home exercise therapy to improve muscle strength and joint flexibility effectively treats pre-radiographic knee OA in community-dwelling elderly: a randomized controlled trial." Clin Rheumatol **38**(1): 133-141.
52. Swain, S., C. Coupland, A. Sarmanova, C. F. Kuo, C. Mallen, M. Doherty and W. Zhang (2023). "Healthcare utilisation and mortality in people with osteoarthritis in the UK: findings from a national primary care database." British Journal of General Practice **73**(733): e615-e622.
53. Unver, B., V. Karatosun and S. Bakirhan (2009). "Reliability of Goniometric Measurements of Flexion in Total Knee Arthroplasty Patients: with Special Reference to the Body Position." Journal of Physical Therapy Science - J PHYS THER SCI **21**: 257-262.
54. White, P. F., O. L. Alvir-Lazo and R. Yumul (2019). Cold laser therapy for acute and chronic pain management: a comparison of low-level and high-intensity laser therapy devices. Anesthesiology News. **Special Edition 2019:** 65.
55. Wickenheisser, V. A., E. M. Zywot, E. M. Rabjohns, H. H. Lee, D. S. Lawrence and T. K. Tarrant (2019). "Laser Light Therapy in Inflammatory, Musculoskeletal, and Autoimmune Disease." Curr Allergy Asthma Rep **19**(8): 37.
56. Wu, M., L. Luan, A. Pranata, J. Witchalls, R. Adams, J. Bousie and J. Han (2022). "Is high intensity laser therapy more effective than other physical therapy modalities for treating knee osteoarthritis? A systematic review and network meta-analysis." Front Med (Lausanne) **9**: 956188.
57. Wu, Y. and F. Zhu (2022). "Effects of transcutaneous electrical nerve stimulation (TENS) in people with knee osteoarthritis: A systematic review and meta-analysis." **36**(4): 472-485.

58. Yang, J., Q. Ji, M. Ni, G. Zhang and Y. Wang (2022). "Automatic assessment of knee osteoarthritis severity in portable devices based on deep learning." J Orthop Surg Res **17**(1): 540.
59. Zamri, N., S. Harith, N. Mat-Hassan and Y. Q. Ong (2021). "Nutritional Status and Health-Related Quality of Life among Knee and Hip Osteoarthritis Patients under Rehabilitation Care in Kuala Nerus, Terengganu, Malaysia." Malays Orthop J **15**(2): 77-88.

UNIVERSITI KEBANGSAAN MALAYSIA
The National University of Malaysia

CENTRE FOR RESEARCH AND INSTRUMENTATION MANAGEMENT

Reference: UKM.PPI.800-1/3/11
Date: 5th October 2023

Dr. Mohd Azzuan Bin Ahmad
Centre for Rehabilitation & Special Needs (ICAREHAB)
Faculty of Health Sciences

Sir/Madam,

APPROVAL OF RESEARCH PROJECT UNDER THE YOUNG RESEARCHER INCENTIVE GRANT (GGPM)

We refer to the above matter.

2. We are pleased to inform you that the Research and Publication Committee Meeting No. 3/2023 on 15th September 2023 has approved your application for the Young Researcher Incentive Grant (GGPM) as per the following details:

- **Project Code**: GGPM-2023-077
- **Project Title**: Disease-Modifying Effects of High-Intensity Laser Therapy as Adjunctive to Rehabilitation Exercise Among Adults with Early Knee Osteoarthritis
- **Approved Allocation**: RM40,000.00
- **Project Duration & Date**: 24 months (01/10/2023 - 30/09/2025)

3. Please note that the approval of this allocation and research project is subject to the following conditions: (i) Research projects approved and receiving funding under the University Research Fund (UP) are not permitted to receive funding from any other agency for the same project. (ii) The project leader and co-researchers must complete the Research Undertaking Letter (SPMP) and submit the original copy to CRIM within 14 days from the date of this letter. (iii) The project status will be activated in the University Research Information System (SMPU) and the Financial System (UFASt) as soon as you submit the completed SPMP.
4. In this regard, you are required to comply with the Guidelines for the Young Researcher Incentive Grant (GGPM) and all regulations and procedures related to university research and financial governance that are currently in force. The use of project allocations must adhere to the approved budget as reflected in SMPU. Vot transfers are allowed only once a year.
5. CRIM congratulates you on this approval. We hope that this achievement will inspire and encourage you to continue contributing to the University's KPIs in research activities, thereby elevating the University's reputation on the international stage.

Thank you.

Director
Centre for Research and Instrumentation Management (CRIM)
Universiti Kebangsaan Malaysia

cc:

- Dean
  Faculty of Health Sciences

UNIVERSITI KEBANGSAAN MALAYSIA
The National University of Malaysia

PUSAT PENGURUSAN PENYELIDIKAN DAN INSTRUMENTASI •
CENTRE FOR RESEARCH AND INSTRUMENTATION MANAGEMENT

Rujukan : UKM.PPI.800-1/3/11
Tarikh : 05 Oktober 2023

Dr. Mohd Azzuan Bin Ahmad
Pusat Kajian Rehabilitasi & Keperluan Khas (ICAREHAB)
Fakulti Sains Kesihatan

Tuan/Puan,

KELULUSAN PROJEK PENYELIDIKAN GERAN GALAKAN PENYELIDIK MUDA (GGPM)

Dengan hormatnya perkara di atas adalah dirujuk.

2. Sukacita dimaklumkan bahawa Mesyuarat Jawatankuasa Penyelidikan dan Penerbitan Bil 3/2023 pada pada 15 September 2023 telah bersetuju meluluskan permohonan tuan/puan bagi Geran Galakan Penyelidik Muda (GGPM) seperti butiran berikut:

Kod Projek	GGPM-2023-077
Tajuk Projek	DISEASE-MODIFYING EFFECTS OF HIGH-INTENSITY LASER THERAPY AS ADJUNCTIVE TO REHABILITATION EXERCISE AMONG ADULTS WITH EARLY KNEE OSTEOARTHRITIS
Peruntukan Diluluskan	RM40,000.00
Tempoh & Tarikh Projek	24 Bulan (01/10/2023 - 30/09/2025)

3. Untuk makluman tuan/puan, kelulusan peruntukan dan projek penyelidikan ini adalah tertakluk kepada perkara berikut:

(i) Projek penyelidikan yang diluluskan dan menerima peruntukan di bawah Dana Universiti Penyelidikan (UP) tidak dibenarkan menerima sebarang peruntukan daripada mana-mana agensi lain bagi projek penyelidikan yang sama;

(ii) Ketua projek dan penyelidik bersama perlu melengkapkan Surat Pengakuan Menjalankan Penyelidikan (SPMP) dan menghantar salinan asal ke CRIM dalam tempoh 14 hari dari tarikh surat ini; dan

(iii) Status projek akan diaktifkan dalam Sistem Maklumat Penyelidikan Universiti (SMPU) dan Sistem Kewangan (UFASt) sebaik sahaja tuan/puan mengemukakan SPMP yang lengkap.

4. Sehubungan dengan ini, tuan/puan dimohon agar dapat mematuhi Garis Panduan Geran Galakan Penyelidik Muda (GGPM) dan semua peraturan serta prosedur berkaitan tadbir urus penyelidikan dan kewangan Universiti yang sedang berkuat kuasa. Penggunaan peruntukan projek adalah berdasarkan belanjawan yang diluluskan seperti mana dalam SMPU. Pemindahan vot hanya dibenarkan sekali setahun sahaja.

5. CRIM mengucapkan tahniah atas kelulusan ini. Semoga kejayaan yang diperoleh ini akan memberikan semangat dan dorongan kepada tuan/puan untuk terus menyumbangkan KPI Universiti dalam aktiviti penyelidikan dan seterusnya menaikkan nama Universiti di persada antarabangsa.

Sekian, terima kasih.

Pengarah
Pusat Pengurusan Penyelidikan dan Instrumentasi (CRIM)
Universiti Kebangsaan Malaysia

sk:
- Dekan
Fakulti Sains Kesihatan

Dokumen ini adalah cetakan komputer dan tidak memerlukan tandatangan

PARTICIPANT INFORMATION SHEET (EXAMPLE)

Research Title:
Photobiomodulation as Adjunctive to Rehabilitation Exercise in Knee Osteoarthritis: A mixed Method Research Design

Introduction:
You are invited to participate in a research study. Before participating in this study, it is important that you take time to read and understand the information in this Information Sheet.

Purpose of Study:
The above study is undertaken to examine the short and long-term effects of high-intensity laser therapy (HILT) combined with rehabilitation exercise on knee pain, physical function, and disability levels among adults with early knee osteoarthritis (KOA) and to investigate the lived experiences and perceptions of individuals with early knee osteoarthritis undergoing the combined intervention of HILT and rehabilitation exercise.

What will the study involve?
Knee osteoarthritis (KOA) is a prevalent chronic condition globally, contributing to knee pain and disability. Recent studies suggest that high-intensity laser therapy (HILT) holds promise as a treatment for KOA. However, clinical evidence supporting its efficacy, particularly over the long term, remains insufficient. This study aims to (i) assess the short- and long-term effects of HILT as a supplement to rehabilitative activities for individuals with mild to moderate KOA, focusing on joint morphologic changes, and (ii) explore the experiences and perceptions of individuals undergoing this combined intervention. The study will employ both quantitative and qualitative methodologies. The first phase of the study will be a randomized double-blinded controlled trial, which will involve 40 participants with mild-to-moderate KOA based on the Kellgren-Lawrence classification. Participants will be randomly assigned to either the HILT with exercise (HILT+E; n=20) or the placebo laser with exercise group (PL+E; n=20). For eight consecutive weeks, both groups will receive their prescribed laser treatment—HILT (5 W, 1064 nm, 19 to 150 J/cm2, and 3190 J each session) or a placebo—along with KOA rehabilitation exercises once a week. Examples of KOA rehabilitation exercises are range of motion, stretching, strengthening, and functional training exercises. The exercise prescription will be modified based on the individual's progression. The outcome measures included are knee X-ray (cartilage thickness and joint space), Knee Injury and Osteoarthritis Outcome Score (KOOS), Timed Up and Go (TUG) test, and Numeric Pain Rating Scale (NPRS), which will be evaluated at three different time points by a blinded assessor: baseline, post-intervention, and follow-up after three months of the last session. Knee X-rays (to evaluate cartilage thickness and joint space) will be taken at baseline and follow-up. Two-way repeated measures ANOVA will be used to assess the effects of time, group, and the interaction between the two groups. In the second phase of the study, structured one-to-one interviews will be carried out with participants to explore patient experiences. Each interview will be audio or video-recorded with participants' consent, and

questions will be posed in a conversational manner to capture both verbal responses and non-verbal cues. The qualitative data will undergo thematic analysis using ATLAS.ti software programs. The findings of this study are pivotal for deepening our understanding of the impacts of HILT and establishing an effective treatment regimen for KOA. These insights have the potential to improve the pathological condition, providing optimism for mitigating KOA progression.

Benefits:

As a participant in the study, you'll contribute to advancing knee osteoarthritis treatment. By undergoing high-intensity laser therapy and rehabilitation exercises, potential benefits include improved knee pain, function, and quality of life. Participation offers access to cutting-edge treatments, personalized care, and monitoring. Your insights will enhance understanding of treatment effectiveness, leading to improved strategies for managing knee osteoarthritis in the future.

Risks:

Participants may experience minor discomfort, adverse reactions like skin irritation or burns, or temporary exacerbation of symptoms during high-intensity laser therapy (HILT) and rehabilitation exercises. Participation requires time commitment and may evoke psychological distress. Confidentiality breaches are possible. Benefits aren't guaranteed. Discuss concerns with the research team before consenting to participation.

Do you have to take part?

Participation in this study is voluntary. If you agree to take part, then you will be asked to sign the "Informed Consent Form". You will be given a copy of the form and this Information Sheet.

Should you decide to participate, you can still withdraw from the study without penalty. Your data will not be used and will be discarded. The researcher may also remove you from the study for a variety of reason. In this event, you will not be penalised or lose your rights as a patient.

Data & Confidentiality:

Information extracted from the study will be interpreted with statistical analysis for testing purposes. The information will be presented as a whole without any specific individuals being mentioned. Your information will be kept private and will not be disclosed to any third party. The data from this study will be made into a report which may be published. Access to the data is only by the research team. The data will be reported in a collective manner with no reference to an individual. Hence your identity will be kept confidential.

Payment and compensation:

You do not have to pay nor will you be paid to participate in this study. You do have to pay for the usual hospital charges.

Who can I ask about the study?

If you have any questions, you can direct them to the research team. You can also contact the REC UKM for clarifications.

Name: Dr. Mohd Azzuan Bin Ahmad
University Lecturer,
Physiotherapy Programme, Centre for Rehabilitation and Special Needs (iCaReHab),
Faculty of Health Sciences, Universiti Kebangsaan Malaysia
Jalan Raja Muda Abdul Aziz, 50300 Kuala Lumpur
Malaysia
Mobile: 012-3297292

Name: Tay Yan Ling
Physiotherapy Master Student,
Faculty of Health Sciences, Universiti Kebangsaan Malaysia
Jalan Raja Muda Abdul Aziz, 50300 Kuala Lumpur
Malaysia
Mobile: 011-55023810

PARTICIPANT INFORMED CONSENT FORM

Research Title: PHOTOBIOMODULATION AS ADJUNCTIVE TO REHABILITATION EXERCISE IN KNEE OSTEOARTHRITIS: A MIXED METHOD RESEARCH DESIGN

Researcher's Name:

I, ……………………………………, Identity No : ……………………......

- have read the information in the Participant Information Sheet **including information regarding the risk in this study.**
- have been given time to think about it and all of my questions have been answered to my satisfaction.
- understand that I may freely choose to withdraw from this study at anytime without reason and without repercussion
- understand that my anonymity will be ensured in the write-up.

I voluntarily agree to be part of this research study, to follow the study procedures, and to provide necessary information to the doctor, nurses, or other staff members, as requested.

……………………………. ………………….
(Signature) (Date)

…………………………….<br>Witness (if any)<br><br>…………………………….<br>(Signature)<br><br>…………………………….<br>(Identity Number)<br><br>…………………………..…<br>(Date)	…………………………….<br>Researcher<br><br>…………………………….<br>(Signature)<br><br>…………………………….<br>(Identity Number)<br><br>…………………………….<br>(Date)

Knee Injury and Osteoarthritis Outcome Score (KOOS)

Source: Roos EM, Roos HP, Lohmander LS, Ekdahl C, Beynnon BD. Knee Injury and Osteoarthritis Outcome Score (KOOS)--development of a self-administered outcome measure. *J Orthop Sports Phys Ther*. 1998 Aug;28(2):88-96.

The Knee Injury and Osteoarthritis Outcome Score (KOOS) is a questionnaire designed to assess short and long-term patient-relevant outcomes following knee injury. The KOOS is self-administered and assesses five outcomes: pain, symptoms, activities of daily living, sport and recreation function, and knee-related quality of life. The KOOS meets basic criteria of outcome measures and can be used to evaluate the course of knee injury and treatment outcome. KOOS is patient-administered, the format is user-friendly and it takes about 10 minutes to fill out.

Scoring instructions

The KOOS's five patient-relevant dimensions are scored separately: Pain (nine items); Symptoms (seven items); ADL Function (17 items); Sport and Recreation Function (five items); Quality of Life (four items). A Likert scale is used and all items have five possible answer options scored from 0 (No problems) to 4 (Extreme problems) and each of the five scores is calculated as the sum of the items included.

Interpretation of scores

Scores are transformed to a 0–100 scale, with zero representing extreme knee problems and 100 representing no knee problems as common in orthopaedic scales and generic measures. Scores between 0 and 100 represent the percentage of total possible score achieved.

Knee Injury and Osteoarthritis Outcome Score (KOOS)

Pain

P1 How often is your knee painful?	☐ Never	☐ Monthly	☐ Weekly	☐ Daily	☐ Always

What degree of pain have you experienced the last week when…?

P2 Twisting/pivoting on your knee	☐ None	☐ Mild	☐ Moderate	☐ Severe	☐ Extreme
P3 Straightening knee fully	☐ None	☐ Mild	☐ Moderate	☐ Severe	☐ Extreme
P4 Bending knee fully	☐ None	☐ Mild	☐ Moderate	☐ Severe	☐ Extreme
P5 Walking on flat surface	☐ None	☐ Mild	☐ Moderate	☐ Severe	☐ Extreme
P6 Going up or down stairs	☐ None	☐ Mild	☐ Moderate	☐ Severe	☐ Extreme
P7 At night while in bed	☐ None	☐ Mild	☐ Moderate	☐ Severe	☐ Extreme
P8 Sitting or lying	☐ None	☐ Mild	☐ Moderate	☐ Severe	☐ Extreme
P9 Standing upright	☐ None	☐ Mild	☐ Moderate	☐ Severe	☐ Extreme

Symptoms

Sy1 How severe is your knee stiffness after first wakening in the morning?	☐ None	☐ Mild	☐ Moderate	☐ Severe	☐ Extreme
Sy2 How severe is your knee stiffness after sitting, lying, or resting later in the day?	☐ None	☐ Mild	☐ Moderate	☐ Severe	☐ Extreme
Sy3 Do you have swelling in your knee?	☐ Never	☐ Rarely	☐ Sometimes	☐ Often	☐ Always
Sy4 Do you feel grinding, hear clicking or any other type of noise when your knee moves?	☐ Never	☐ Rarely	☐ Sometimes	☐ Often	☐ Always
Sy5 Does your knee catch or hang up when moving?	☐ Never	☐ Rarely	☐ Sometimes	☐ Often	☐ Always
Sy6 Can you straighten your knee fully?	☐ Always	☐ Often	☐ Sometimes	☐ Rarely	☐ Never
Sy7 Can you bend your knee fully?	☐ Always	☐ Often	☐ Sometimes	☐ Rarely	☐ Never

Activities of daily living

What difficulty have you experienced the last week…?

A1 Descending	☐ None	☐ Mild	☐ Moderate	☐ Severe	☐ Extreme
A2 Ascending stairs	☐ None	☐ Mild	☐ Moderate	☐ Severe	☐ Extreme
A3 Rising from sitting	☐ None	☐ Mild	☐ Moderate	☐ Severe	☐ Extreme
A4 Standing	☐ None	☐ Mild	☐ Moderate	☐ Severe	☐ Extreme
A5 Bending to floor/picking up an object	☐ None	☐ Mild	☐ Moderate	☐ Severe	☐ Extreme
A6 Walking on flat surface	☐ None	☐ Mild	☐ Moderate	☐ Severe	☐ Extreme
A7 Getting in/out of car	☐ None	☐ Mild	☐ Moderate	☐ Severe	☐ Extreme
A8 Going shopping	☐ None	☐ Mild	☐ Moderate	☐ Severe	☐ Extreme
A9 Putting on socks/stockings	☐ None	☐ Mild	☐ Moderate	☐ Severe	☐ Extreme
A10 Rising from bed	☐ None	☐ Mild	☐ Moderate	☐ Severe	☐ Extreme
A11 Taking off socks/stockings	☐ None	☐ Mild	☐ Moderate	☐ Severe	☐ Extreme
A12 Lying in bed (turning over, maintaining knee position)	☐ None	☐ Mild	☐ Moderate	☐ Severe	☐ Extreme
A13 Getting in/out of bath	☐ None	☐ Mild	☐ Moderate	☐ Severe	☐ Extreme
A14 Sitting	☐ None	☐ Mild	☐ Moderate	☐ Severe	☐ Extreme
A15 Getting on/off toilet	☐ None	☐ Mild	☐ Moderate	☐ Severe	☐ Extreme
A16 Heavy domestic duties (shovelling, scrubbing floors, etc)	☐ None	☐ Mild	☐ Moderate	☐ Severe	☐ Extreme
A17 Light domestic duties (cooking, dusting, etc)	☐ None	☐ Mild	☐ Moderate	☐ Severe	☐ Extreme

Sport and recreation function

What difficulty have you experienced the last week…?

Sp1 Squatting	☐ None	☐ Mild	☐ Moderate	☐ Severe	☐ Extreme
Sp2 Running	☐ None	☐ Mild	☐ Moderate	☐ Severe	☐ Extreme
Sp3 Jumping	☐ None	☐ Mild	☐ Moderate	☐ Severe	☐ Extreme
Sp4 Turning/twisting on your injured knee	☐ None	☐ Mild	☐ Moderate	☐ Severe	☐ Extreme
Sp5 Kneeling	☐ None	☐ Mild	☐ Moderate	☐ Severe	☐ Extreme

Knee-related quality of life

Q1 How often are you aware of your knee problems?	☐ Never	☐ Monthly	☐ Weekly	☐ Daily	☐ Always
Q2 Have you modified your lifestyle to avoid potentially damaging activities to your knee?	☐ Not at all	☐ Mildly	☐ Moderately	☐ Severely	☐ Totally
Q3 How troubled are you with lack of confidence in your knee?	☐ Not at all	☐ Mildly	☐ Moderately	☐ Severely	☐ Totally
Q4 In general, how much difficulty do you have with your knee?	☐ None	☐ Mild	☐ Moderate	☐ Severe	☐ Extreme

WHO-5 Well-being Index

Please respond to each item by marking <u>one box per row,</u> regarding how you felt in the last two weeks.		All of the time	Most of the time	More than half the time	Less than half the time	Some of the time	At no time
WHO 1	I have felt cheerful in good spirits.	☐ 5	☐ 4	☐ 3	☐ 2	☐ 1	☐ 0
WHO 2	I have felt calm and relaxed.	☐ 5	☐ 4	☐ 3	☐ 2	☐ 1	☐ 0
WHO 3	I have felt active and vigorous.	☐ 5	☐ 4	☐ 3	☐ 2	☐ 1	☐ 0
WHO 4	I woke up feeling fresh and rested.	☐ 5	☐ 4	☐ 3	☐ 2	☐ 1	☐ 0
WHO 5	My daily life has been filled with things that interest me.	☐ 5	☐ 4	☐ 3	☐ 2	☐ 1	☐ 0

Scoring:
The raw score is calculated by totaling the figures of the five answers. The raw score ranges from 0 to 25, 0 representing worst possible and 25 representing best possible quality of life.

To obtain a percentage score ranging from 0 to 100, the raw score is multiplied by 4. A percentage score of 0 represents worst possible, whereas a score of 100 represents best possible quality of life.

Regional Office for Europe WHO. Use of Well-Being Measures in Primary Health Care - The DepCare Project. Health for All, Target 12, 1998 [http://www.who.dk/document/e60246.pdf]

Bech P. Measuring the dimensions of psychological general well-being by the WHO-5. QoL Newsletter 2004; 32: 15-16.

36-Item Short Form Survey Instrument (SF-36)

RAND 36-Item Health Survey 1.0 Questionnaire Items

Choose one option for each questionnaire item.

1. In general, would you say your health is:

- 1 - Excellent
- 2 - Very good
- 3 - Good
- 4 - Fair
- 5 - Poor

---

2. **Compared to one year ago**, how would you rate your health in general **now**?

- 1 - Much better now than one year ago
- 2 - Somewhat better now than one year ago
- 3 - About the same
- 4 - Somewhat worse now than one year ago
- 5 - Much worse now than one year ago

The following items are about activities you might do during a typical day. Does **your health now limit you** in these activities? If so, how much?

	Yes, limited a lot	Yes, limited a little	No, not limited at all
3. **Vigorous activities**, such as running, lifting heavy objects, participating in strenuous sports	◯ 1	◯ 2	◯ 3
4. **Moderate activities**, such as moving a table, pushing a vacuum cleaner, bowling, or playing golf	◯ 1	◯ 2	◯ 3
5. Lifting or carrying groceries	◯ 1	◯ 2	◯ 3
6. Climbing **several** flights of stairs	◯ 1	◯ 2	◯ 3
7. Climbing **one** flight of stairs	◯ 1	◯ 2	◯ 3
8. Bending, kneeling, or stooping	◯ 1	◯ 2	◯ 3
9. Walking **more than a mile**	◯ 1	◯ 2	◯ 3
10. Walking **several blocks**	◯ 1	◯ 2	◯ 3
11. Walking **one block**	◯ 1	◯ 2	◯ 3
12. Bathing or dressing yourself	◯ 1	◯ 2	◯ 3

During the **past 4 weeks**, have you had any of the following problems with your work or other regular daily activities **as a result of your physical health**?

	Yes	No
13. Cut down the **amount of time** you spent on work or other activities	◯ 1	◯ 2
14. **Accomplished less** than you would like	◯ 1	◯ 2
15. Were limited in the **kind** of work or other activities	◯ 1	◯ 2
16. Had **difficulty** performing the work or other activities (for example, it took extra effort)	◯ 1	◯ 2

During the **past 4 weeks**, have you had any of the following problems with your work or other regular daily activities **as a result of any emotional problems** (such as feeling depressed or anxious)?

	Yes	No
17. Cut down the **amount of time** you spent on work or other activities	◯ 1	◯ 2
18. **Accomplished less** than you would like	◯ 1	◯ 2
19. Didn't do work or other activities as **carefully** as usual	◯ 1	◯ 2

20. During the **past 4 weeks**, to what extent has your physical health or emotional problems interfered with your normal social activities with family, friends, neighbors, or groups?

◯ 1 - Not at all

◯ 2 - Slightly

◯ 3 - Moderately

◯ 4 - Quite a bit

◯ 5 - Extremely

21. How much **bodily** pain have you had during the **past 4 weeks**?

- 1 - None
- 2 - Very mild
- 3 - Mild
- 4 - Moderate
- 5 - Severe
- 6 - Very severe

22. During the **past 4 weeks**, how much did **pain** interfere with your normal work (including both work outside the home and housework)?

- 1 - Not at all
- 2 - A little bit
- 3 - Moderately
- 4 - Quite a bit
- 5 - Extremely

These questions are about how you feel and how things have been with you **during the past 4 weeks**. For each question, please give the one answer that comes closest to the way you have been feeling.

How much of the time during the **past 4 weeks**...

	All of the time	Most of the time	A good bit of the time	Some of the time	A little of the time	None of the time
23. Did you feel full of pep?	◯ 1	◯ 2	◯ 3	◯ 4	◯ 5	◯ 6
24. Have you been a very nervous person?	◯ 1	◯ 2	◯ 3	◯ 4	◯ 5	◯ 6
25. Have you felt so down in the dumps that nothing could cheer you up?	◯ 1	◯ 2	◯ 3	◯ 4	◯ 5	◯ 6
26. Have you felt calm and peaceful?	◯ 1	◯ 2	◯ 3	◯ 4	◯ 5	◯ 6
27. Did you have a lot of energy?	◯ 1	◯ 2	◯ 3	◯ 4	◯ 5	◯ 6
28. Have you felt downhearted and blue?	◯ 1	◯ 2	◯ 3	◯ 4	◯ 5	◯ 6
29. Did you feel worn out?	◯ 1	◯ 2	◯ 3	◯ 4	◯ 5	◯ 6
30. Have you been a happy person?	◯ 1	◯ 2	◯ 3	◯ 4	◯ 5	◯ 6
31. Did you feel tired?	◯ 1	◯ 2	◯ 3	◯ 4	◯ 5	◯ 6

---

32. During the **past 4 weeks**, how much of the time has **your physical health or emotional problems** interfered with your social activities (like visiting with friends, relatives, etc.)?

◯ 1 - All of the time

◯ 2 - Most of the time

◯ 3 - Some of the time

◯ 4 - A little of the time

◯ 5 - None of the time

How TRUE or FALSE is **each** of the following statements for you.

	Definitely true	Mostly true	Don't know	Mostly false	Definitely false
33. I seem to get sick a little easier than other people	○ 1	○ 2	○ 3	○ 4	○ 5
34. I am as healthy as anybody I know	○ 1	○ 2	○ 3	○ 4	○ 5
35. I expect my health to get worse	○ 1	○ 2	○ 3	○ 4	○ 5
36. My health is excellent	○ 1	○ 2	○ 3	○ 4	○ 5

ABOUT

The RAND Corporation is a research organization that develops solutions to public policy challenges to help make communities throughout the world safer and more secure, healthier and more prosperous. RAND is nonprofit, nonpartisan, and committed to the public interest.

1776 Main Street
Santa Monica, California 90401-3208

RAND® is a registered trademark. Copyright © 1994-2016 RAND Corporation.
